# SeismoQuakeGNN: a hybrid framework for spatio-temporal earthquake prediction with transformer-enhanced models

**DOI:** 10.3389/frai.2025.1690476

**Published:** 2025-12-02

**Authors:** Anny Leema, Ponnuraman Balakrishnan, Gladys Gnana Kiruba, Ganesarathinam Rajarajan, Stuti Goel, Prisha Aggarwal

**Affiliations:** School of Computer Science and Engineering, Vellore Institute of Technology, Vellore, India

**Keywords:** seismic data, random forest regression, graph neural network, Long Short-Term Memory, SeismoQuakeGNN

## Abstract

Accurate predictions of earthquakes are crucial for disaster preparedness and risk mitigation. Conventional machine learning models like Random Forest, SVR, and XGBoost are frequently used for seismic forecasting; however, capturing the intricate spatiotemporal relationships in earthquake data remains a challenge. To overcome this issue, we propose SeismoQuakeGNN, a novel Graph Neural Network (GNN) and Transformer-based hybrid framework that integrates spatial and temporal learning for improved seismic forecasting. Unlike existing GNN-based models, SeismoQuakeGNN introduces an optimized spatial encoding mechanism to dynamically learn seismic interdependencies, coupled with a Transformer-driven attention module to capture long-range temporal correlations. Furthermore, initial experiments with XGBoost demonstrated its limitations in learning earthquake patterns, reinforcing the need for deep spatial–temporal modeling. The new SeismoQuakeGNN method is capable of substantial and efficient data processing of relationships in both space and time, as well as providing superior transfer to different seismic areas, thereby qualifying as a dependable starting point to extensive earthquake forecasting and hazard evaluation.

## 1 Introduction

The most terrible natural disasters are earthquakes, which are responsible for a large number of deaths, the destruction of infrastructure, and disruptions in the economy. The ability to forecast earthquakes is one of the most proactive and effective ways of minimizing their adverse effects and preparing communities to cope with them. One of the major challenges in forecasting the severity of earthquakes is that the relationships between geological and seismic parameters are very complex, and the properties of the earth are highly non-linear, dynamic, and complex. Ground backfill materials have been tested for performance under repeated heating around buried cables, and this has created a demand for models that can accurately simulate the time-dependent changes in these materials. These soil-heat exchanges have been one of the factors to consider in the establishment of forecast systems that are not only adaptable to changes in time but also to different surroundings, which are similar to the changes that occur instantly the case of seismic areas (Ahmad et al., 2019; Ahmad et al., 2025b; Ahmad et al., 2021). In fact, one of the most important physical criteria among the geophysical parameters to be recorded is the timeliness of the occurrence of the earthquake, which can also be characterized by longitudes, latitudes, depths, the geological setting, and dmin (minimum distance to the nearest seismic station). Parameters such as these are predictors that make it possible to identify the relationship between seismic activity and magnitude. Modern computational methods, including but not limited to machine learning (ML) and deep learning (DL) algorithms, paved the way for precision and better reliability in predicting the magnitude of earthquakes. AI-driven predictive diagnostics, successfully applied in domains such as corrosion monitoring of clay soils, demonstrate the potential of cross-domain learning to strengthen earthquake forecasting frameworks (Ahmad et al., 2025a). Traditional statistical methods have the ability, on the one hand, to reveal valuable insights, but, on the other hand, they lack the capacity to truly depict the subtle character of these features in a very. On the other hand, use hidden patterns and non-linear correlations to make predictions more accurate. Random Forest, Support Vector Regression (SVR), and XGBoost were employed to prepare the prediction models. Among them, methods were particularly selected for the sake of considerable records of success in analyzing both linear and non-linear correlations on structured datasets. Additionally, algorithms such as Feedback Neural Networks and Transformer algorithms were employed to train on the fault cuts’ spatial and temporal patterns. The target function of this deep learning network, which is known for its usual variety of data in various domains, is now used for obtaining the prediction of the earthquake magnitude more precisely.

Feedback Neural Networks (FNNs) and transformer algorithms are used to recognize more complex spatial and temporal patterns in data. These sophisticated techniques, having first been developed for use in various fields, are applicable in seismic datasets, which contribute to an increased number of correct earthquake magnitude predictions. The research activities included preliminary figures preprocessing and modification of the seismic data that will subsequently play such a role to make it fit and harmonious with the various algorithms being used.

## 2 Related work

Recent advancements in AI and ML have significantly boosted earthquake prediction abilities, focusing on both accuracy and efficiency. The estimation of the intensity of an earthquake was one example where Support Vector Machines (SVM) with hyperparameter optimization were implemented. The authors reveal the capabilities of AI to be able to prepare better for future disasters (Ahmed et al., 2024). In a similar vein, a real-time AI framework that combines IoT devices with edge and cloud computing was designed. The system demonstrated an accuracy of more than 90%, as a result of which it was found that the combination of these technologies for field data handling and decision-making is simply feasible and efficient (Bhatia et al., 2023).

Bowie State University (2024) has brought the changes by incorporating seismic data from drones and satellites, thereby succeeding in the prediction of seismic phases with the help of AI, as well as forecasting the intensity of seismic events with a high degree of accuracy. A predictive model was developed forecasting the magnitudes of large earthquakes in Taiwan using deep learning methodologies (Huang et al., 2018). Meanwhile, Akhoondzadeh (2024) emphasized the role of satellite data for anomaly detection in earthquake prediction, though challenges in achieving low-uncertainty warnings persist. Deep learning approaches have demonstrated significant promise. Convolutional Neural Networks (CNNs) are employed to predict large earthquakes in Taiwan, achieving notable improvements over traditional methods. Vasti and Dev (2024) expanded this with Long Short-Term Memory (LSTM) networks, which showed superior accuracy in magnitude prediction while reducing computational complexity. Additionally, Banna et al. (2021) and Banna et al. (2020) introduced an attention-based Bi-LSTM network, achieving remarkable precision in predicting earthquake occurrences and locations. Hybrid models have also proven effective in earthquake hazard estimation. A combination of Inception v3 and XGBoost models is utilized along with explainable AI techniques to estimate spatial probability and hazard in the Arabian Peninsula, highlighting the importance of integrating geophysical and seismological factors (Jena et al., 2023). Similarly, various machine learning algorithms are compared, and the highest accuracy is achieved with Random Forest for predicting major earthquake events (Mallouhy et al., 2019). The researchers focus on the ionosphere, a layer of the Earth’s atmosphere that is influenced by various factors, including seismic activity. They hypothesize that changes in the ionosphere, such as fluctuations in electron density or temperature, may serve as precursors to earthquakes (Zhou et al., 2020). For real-time applications, a danger theory-based model is introduced using artificial immunity, achieving superior prediction accuracy across multiple seismic indicators (Akyol et al., 2020). The prediction of the earthquake’s magnitude in the Hindukush area was the main focus of this study, where Machine Learning was used, and the writers made the calculation of eight seismic indicators from the historical earthquake dataset of the region. These indicators come from basic geophysical concepts such as the Gutenberg-Richter law, characteristic earthquake magnitudes, and the decrease of seismic activity (Asim et al., 2016).

Several machine learning approaches, namely, Artificial Neural Networks (ANN), Random Forest (RF), and Support Vector Machine (SVM), were explored to foresee major earthquakes in the seismically prolific North Zagros region. The results are clearly awarded to the ANN model as it displays the best skills in predicting significant seismic events (Ommi and Hashemi, 2024). A recently designed deep learning model, which uses a transformer, has been implemented to forecast the intensity of earthquakes in the Horn of Africa (Kassie et al., 2023). The study reports that the P-Alert system, a low-cost early earthquake warning system, was indeed effective during the 6.4-magnitude earthquake in Hualien, Taiwan, in 2018. Through the research, the productive nature of the system regarding instant map creation and improvement of the robustness of the real-time seismic hazard mitigation was underlined (Wu et al., 2018). The authors trained a somewhat energized model, a convolutional neural network (CNN)-based algorithm called DPick, and this model was trained with data containing 17,717 accelerograms to be able to identify and recognize the characteristics of primary wave arrivals (Yanwei et al., 2021).

Deep learning architecture, in particular, an LSTM network was applied to predict spectral accelerations or the ground motion of aftershocks. In addition to information about the main shock’s magnitude, location, and the subsequent aftershock sequences, a dataset of historical earthquake records was included to train the model. The trained model was then used to predict the spectral accelerations of future aftershocks at different locations and at different frequencies (Ding et al., 2021). Amongst the latest research, a new approach to deep learning and hybrid models has also been highlighted. Auto-regressive forecasting techniques such as LSTM, Temporal Convolutional Networks (TCN), and Transformers have given higher accuracy for laboratory earthquake predictions (Laurentia et al., 2022). Several ML models were tested by the researchers, and Polynomial Regression was found to be the most efficient for the prediction of magnitude, while Random Forest was the most effective in predicting depth (Yanwei et al., 2021). Some researchers chose to concentrate on CNN and LSTM models for earthquake magnitude prediction, which led to significant efficiency gains (Alarifi et al., 2012; Kassie et al., 2023). The evaluation of several ML algorithms with ensemble methods showed that the more accurate results were obtained through those methods, thus further strengthening the case of ensemble approaches in seismic applications (Mallouhy et al., 2019). A comprehensive look into the use of machine learning in earthquake prediction was the focus of the 2024 survey. Many efforts in this branch of seismology that used machine learning methods to search seismic data for patterns and find a way to predict future earthquakes in the study were examined. The datasets used, the features exploited, the magnitude of completeness that were suitable for consideration, and the performance metrics that they used in these studies were reviewed (Galkina and Grafeeva, 2019). A review was undertaken on the use of AI and IoT in earthquake prediction. The review, in the main, talks about the ML-based models and IoT-based technologies used for earthquake forecasting, along with the limits of the recent solutions and open research avenues. To boot, the examined study brought up some insights on potential methods and solutions for overcoming earthquake prediction bottlenecks based on the learned experiences in AI and IoT in varied areas (Pwavodi et al., 2024a).

A hybrid of RNN, PRNN, Random Forest, and LPB was used to make earthquake predictions in the Hindu Kush region, with the result of different models’ high precision, accuracy, and recall (Asim et al., 2020). Deep Learning Models with dynamic loss functions are powerful when trained on a variety of regions compared with conventional methodologies on the map and Root Mean Squared Error (RMSE) and Mean Absolute Error (MAE) scores (Li W. et al., 2020). An enhanced Fuzzy Hybrid Machine Learning Model [Flower Pollination Algorithm (FPA) with Least Squares Support Vector Machine (LS-SVM)] that was applied on the seismological datasets from different areas gave promising accuracy results, leading to the conclusion that the tested method outperformed conventional ones on the basis of RMSE, MAE, Percent Mean Relative Error (PMRE), and Symmetric Mean Absolute Percentage Error (SMAPE) metrics (Salam et al., 2020). In the case of India, Simple Logistic, Random Forest, and Logistic Regression were employed for the evaluation of its predictive power in terms of the number of events predicted in six different regions and the results have shown a high level of consistency (in terms of accuracy) as a predictor of earthquakes (Debnath et al., 2020). Moreover, ANN, Random Tree, CHAID, Discriminant models, XGBoost Tree, and Tree-AS have been tested on six datasets across various regions in India, with XGBoost tree and ANN (Chittora et al., 2020a). For large-scale earthquake monitoring, an AdaBoost-based ensemble model has been employed using satellite data from 1,371 earthquakes (2006–2013), yielding promising results with good accuracy, precision, and recall (Xiong et al., 2020). In a systematic review of 31 studies on earthquake prediction from 2017 to 2021, various machine learning algorithms have been assessed, showing significant promise in earthquake forecasting (Ridzwan and Yusoff, 2023). Deep learning approaches such as convolutional neural networks (CNN), LSTM < ARIMA, and singular spectrum analysis (SSA) have been compared using monthly earthquake magnitude data from Turkey, with deep learning outperforming traditional statistical models based on RMSE and MAE (Çekim et al., 2023). The incorporation of waveform and text-based multimodal deep learning networks has further improved earthquake magnitude predictions using seismic data from multiple seismometers. These models achieve RMSE < 0.38, MAE < 0.29 after 3 s, stabilizing to RMSE 0.20, MAE 0.15 after 14 s, demonstrating reasonable precision in real-time applications (Hou et al., 2024). A broader application of machine learning techniques across various seismic datasets has confirmed their effectiveness in earthquake seismology, reinforcing their utility in hazard prediction and early warning systems (Kubo S. et al., 2024). XGBoost, Stacking Regressor, and LSTM models have been tested on seismic data from Düzce, Turkey, with XGBoost achieving the lowest MAE and RMSE among the evaluated models (Dikmen, 2024).

After looking deeper into various models of machine learning and deep learning, it was verified that one of the major success stories of the scientific field was earthquake seismology, where, primarily in terms of the accuracy of predictions and the ability to detect early signals, this technology proved to be significantly effective (Kubo H. et al., 2024). The applications of ML and artificial AI in the analysis of seismic signals, complemented by the Internet of Things (IoT), have been shown to achieve an accuracy rate of over 90%, proving the capability of these technologies in predictive seismic analysis (Pwavodi et al., 2024b). Furthermore, advancements in the field of artificial intelligence have allowed the study of electromagnetic signals for the first time using data from Los Angeles, which has resulted in a more accurate prediction of earthquake magnitudes in this city (Yavas et al., 2024).

One of the ways to use deep learning for better detection of earthquakes is to use the data collected from a variety of locations to look for a pattern of activity that can be used to detect earthquakes. In this way, the researchers managed to increase the detection rate while also minimizing false alarms (Sun et al., 2024). The authors have presented a series of validation tests that have demonstrated the performance of the proposed classification, curve fitting, and machine learning-based neural network models applied to seismic datasets from multiple localities to forecast earthquakes (Chittora et al., 2020b). Besides, different machine learning models have been applied with success to learn seismic activity, describing complex nonlinear interactions between the features and the magnitudes of earthquakes (Hu et al., 2024; Yousefzadeh et al., 2021).

Such deep learning models that are designed for and have been trained on real-time field-collected seismic data have been repeatedly confirmed to lead to better results as compared to traditional methods for estimating earthquake damage (Kizilay et al., 2024). In addition, the fusion of earthquake data from both seismic and non-seismic activities together with machine learning tools such as Support Vector Machines (SVM) and Random Forest has resulted in a more reliable, non-linear, and dynamic quake forecast that features a greater accuracy rate (Abdalzaher, 2024). Early detection relying on IoT devices, combined with cloud computing, has been a significant factor in contributing to higher accuracy, leading to effective prevention of the hazards of earthquakes (Venkatanathan, 2024).

Machine learning for earthquakes and different methods of machine learning for earthquakes have been thoroughly compared, acknowledging their advantages and the difficulty in predicting earthquakes (Galkina and Grafeeva, 2019). The implementation of an IoT-based system has not only enhanced the accuracy of seismic activity forecasts but has also allowed for the creation of early earthquake warning systems (Abdalzaher et al., 2023; Kolivand et al., 2024). An expert review compared the prediction models and found that the problem of predicting the time, place, and magnitude of earthquakes can be done in a reliable way is the main challenge (Galkina and Grafeeva, 2019). There have been some comprehensive reports on integrating the use of DL in earthquake engineering to demonstrate the range of technical changes and challenges mainstream seismic models may represent (Rosca, 2024; Ajai, 2024). The Earthquake Transformer, a deep learning model relying on the Transformer architecture, has been trained on the offshoot of the global seismic wave database, and, much more evident than with conventional models, the great pre-training accuracy with fewer false recognition possibilities was the major attribute identified (Xie, 2024). AI has optimized prediction accuracy to an extent that has made the use of AI techniques in the analysis of seismic data a phenomenon that calls for further investigations (Abdalzaher et al., 2024). Lastly, DNNs (Deep Neural Networks) have been employed in the classification of seismic waveforms, leading to a better earthquake early warning system in terms of accuracy (Mousavi et al., 2020a). Surface response models relying on machine learning methods have been created for seismic activities; thus, the use of high-accuracy forecasts of the flexural strengths of structures under an earthquake was successfully predicted (Panakkat and Adeli, 2007; Apriani et al., 2021). Several ML models have been validated with earthquake datasets, which, through the extraction of advanced features such as waveforms, forecasted the seismic ground motion with very high accuracy (Afshar et al., 2024; Mondol, 2021b). The Transformer architecture was introduced, where sequence-to-sequence learning is achieved using self-attention mechanisms instead of recurrent or convolutional structures, resulting in faster training and improved performance (Vaswani et al., 2017). To conclude, the employment of neural networks in the analysis of seismic events has confirmed their great value in forecasting the earthquake magnitude with high classification accuracy (Joshi, 2024).

### 2.1 Challenges in current methods

Earthquake forecasting is an extremely intricate area where, by nature, one has to come up with models that can efficiently represent the Earth’s ever-changing, interconnected processes beneath the crust. Existing techniques suffer from a range of problems, for instance, requiring that one handle high-dimensional spatio-temporal dependencies, inadequate data, and the range of data sources. The earthquakes happening on the Earth’s surface are the result of nonlinear interactions between crust, mantle, and tectonic plates, which are moving relative to each other. Traditional methods frequently rest on several assumptions that are not always justified, such as neglecting the time sequence of seismic events and the spatial dependencies between fault lines. Multiple models have difficulty in catching long-term dependencies and do not integrate temporal dynamics with spatial data successfully. Seismic datasets are often inadequately populated and only specific to the region. Checks are carried out in places like Japan and California, using historical data to get a better understanding of earthquake patterns, while in other areas that are prone to earthquakes, there is not enough labeled data to train machine learning models. Little data may prevent traditional as well as deep learning models from mastering the generalization to different areas. Mostly, the models that have been developed to date are those that only have information for one particular type of data, which limits their capacity to accommodate multi-modal inputs and thereby derive meaningful correlations. Table 1 summarizes the current earthquake prediction methods that face problems in traditional, machine learning, and deep learning approaches.

**Table 1:** Challenges in traditional, machine learning, and deep learning-based earthquake prediction methods.

Category	Research work	Challenges identified	Approach used	Limitations
Statistical Models	Gutenberg and Richter (1954)	Simple regression fails to capture spatio-temporal complexities.	Linear regression on seismic activity.	Oversimplifies non-linear dependencies.
Machine Learning Models	Mousavi et al. (2019)	Difficulty generalizing due to limited labeled data.	Random Forest and Support Vector Machines.	Requires feature engineering; struggles with multi-modal data.
Deep Learning (CNNs)	Perol et al. (2018)	Inability to capture long-term temporal dependencies.	Convolutional Neural Networks for spatial feature extraction.	Cannot model sequential dependencies effectively.
Recurrent Neural Networks	Mousavi et al. (2020b) and Suwal and Yen (2021)	Handles time series but ignores spatial interactions.	RNN-based time-series modeling.	Poor scalability with high-dimensional spatio-temporal data.
Graph Neural Networks	Zhang et al. (2020)	Models spatial relationships but lacks temporal integration.	Graph Neural Networks on fault maps.	Focuses only on spatial interactions; does not incorporate waveform or geological data.
Multi-Modal Learning	Wang et al. (2021)	Challenges in fusing heterogeneous data.	Early fusion using concatenated features.	Loss of intricate interdependencies during data fusion.
Transformer Models	González et al. (2019)	High computational cost; limited real-world applications.	Attention mechanisms for spatial and temporal learning.	Requires more optimization and exploration in the seismic prediction domain.

Deep learning and machine learning architectures have made a significant step forward in their functionalities to forecast earthquakes and to analyze seismic events. This fact is clearly demonstrated in the studies that were reviewed. The researchers have successfully applied CNNs to foresee the spectral accelerations of the aftershock ground motions, and in their recent study, they have stated that CNNs are proceeding beyond the traditional approach (Ding et al., 2021). In the same manner, González et al. (2019) implemented LSTM networks to forecast the magnitude of the earthquake based on the seismic time series data, and the result was that the accuracy of the suggested method was higher than that of the conventional models. The different experiments have been focused on mixed architecture consisting of Support Vector Regressor (SVR) and Recurrent Neural Networks (RNNs) for the purpose of better earthquake prediction. These studies underscore the potential of neural networks in detecting and evaluating the seismic hazard, and thus LSTMs and other recurrent models are more successful in this regard due to temporal dependency (Asim et al., 2018). Besides that, some studies conducted investigations on neural networks and mixed models for earthquake-related forecasting in world scenarios such as ground motion, seismic response, and shaking intensity. For instance, ANN has been used for the prediction of ground motion, which demonstrated more accuracy than the conventional regression models (Li Y. et al., 2020). The use of CNNs was to predict the effect of an earthquake on the ground directly from the waveform, thus enabling better performance of the established ground motion prediction equations. The introduction of a neural network model for the seismic response pointed out the flexibility of machine learning in the case of actual engineering. The implementation of machine learning and artificial intelligence continues to grow in the seismic domain, and the instruments they provide are very useful as pre-alert systems, risk assessment, and disaster preparedness (Dhanya and Raghukanth, 2017).

## 3 Proposed system design

**Figure 1:**
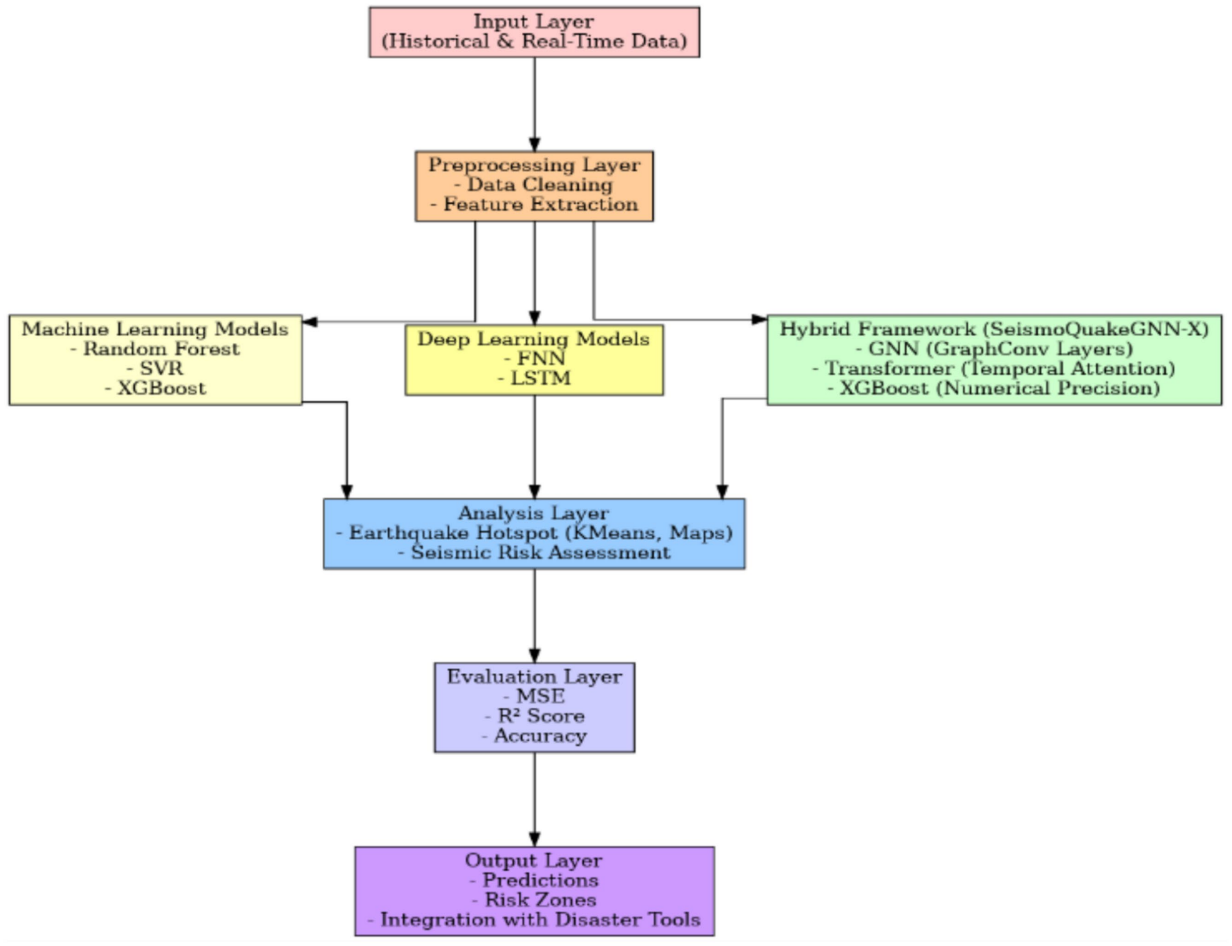
Multi-layered framework for seismic risk prediction.

One of the main topics of our research is the prediction and assessment of seismic risks. We have designed a comprehensive framework that is shown through a multi-layered architecture in Figure 1, which employs (ML), deep learning (DL), and hybrid modeling methods for understanding seismic data by easy mapping of areas with different risk levels and accessing data by disaster relief workers, besides predicting an earthquake. Comparable lattice-based modeling frameworks have been applied successfully to complex geomaterials, validating the role of advanced numerical methods in capturing spatial dependencies (Rizvi et al., 2020). The suggested framework for the prediction of seismic risk relies on a unified pipeline that combines historical and real-time data. Earthquake catalogs and sensor data are aspects of the Input Layer, which are cleaned and processed by the preprocessing layer for delivering features such as magnitudes and epicenter coordinates that are understandable to the next layer. Layer of modeling is a blend of machine learning (Random Forest, SVR, XGBoost), deep learning (FNN, LSTM, GCN, GAT, GraphSAGE), and a Hybrid Framework (SeismoQuakeGNN with GNN and transformer) to figure out spatial–temporal dependencies and thus enhance predictive accuracy. Clustering is used to search for quake hotspots in the analysis layer, giving a seismic risk evaluation and also generating visualizations for decision-making. A metric system, including MSE, *R*^2^ Score, and Accuracy, is used in the evaluation stage to set the performance level. Output Layer, in fact, pulls together predictions, risk zones, and actionable insights, and hence, the information is integrated with disaster management tools, thus enhancing preparedness and mitigation.

**Figure 2:**
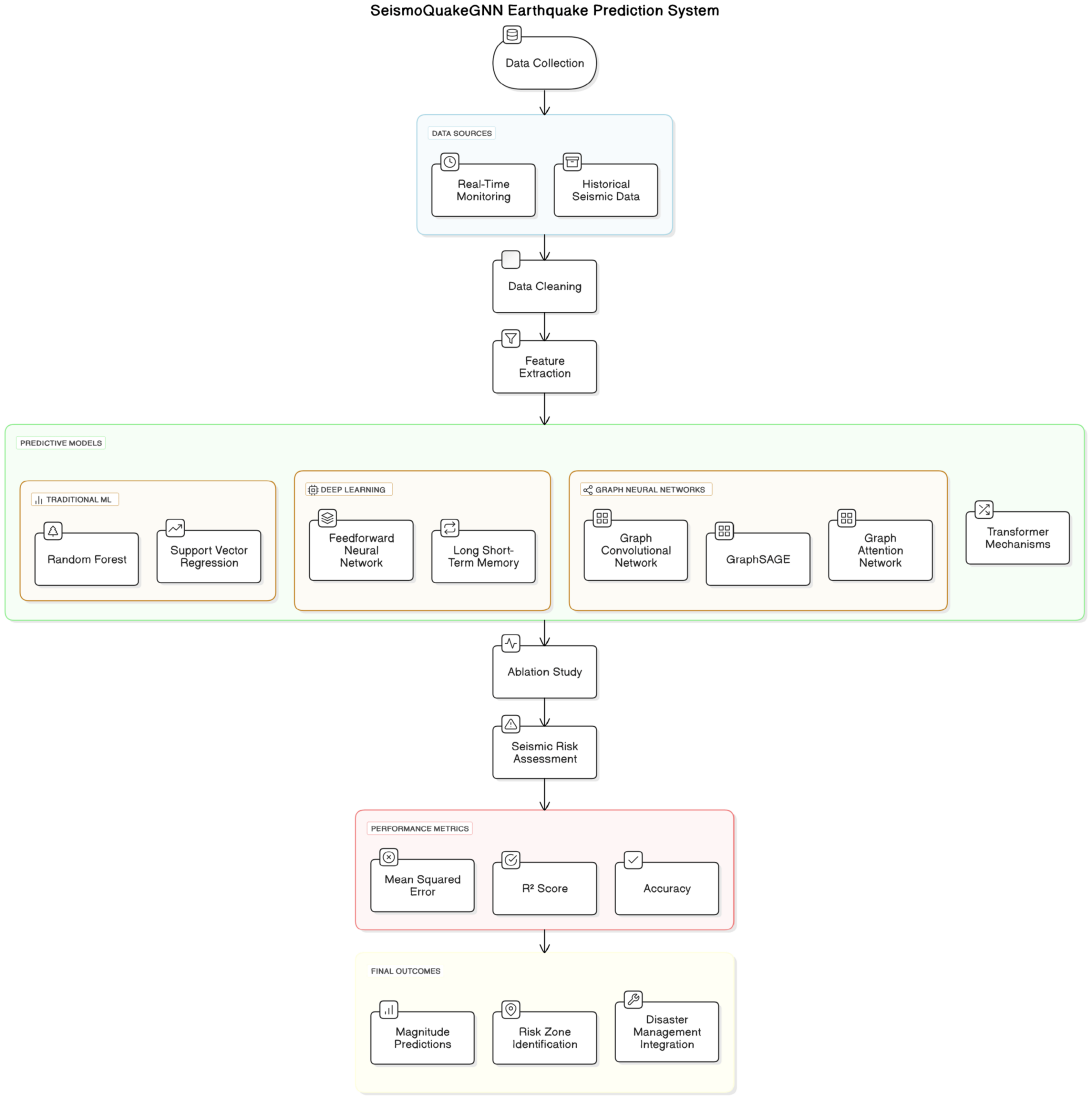
SeismoQuakeGNN earthquake prediction system architecture.

Figure 2 offers a clean and concise overview when compared to the previous chart, highlighting the contributions that each modeling component has made. The process of ‘data collection’ is started, in which the two most significant sources—real-time earthquake monitoring data and historical seismic data—are incorporated. The real-time aspect of the monitoring is in charge of the latest seismic activity, whereas the historical data is used to understand the long-term patterns. These data are then being removed from errors and noise, and feature extraction is followed, wherein the main parameters such as latitude, longitude, depth, magnitude, and other seismic attributes are chosen for model training.

Next, the extracted features are multiplied by different predictive models. Regular ML approaches, such as Random Forest (RF) and Support Vector Regression (SVR), use the logical structure of seismic data to analyze it in terms of patterns. The deep learning models, such as FNNs (Feedforward Neural Networks) and LSTM (Long Short-Term Memory) networks, are devoted to mastering the nonlinear and dependent relationships in sequentialization. Graph Neural Networks (GNNs) are made for the specific duty of visualizing the links among seismic events, which is done by modeling earthquakes as interconnected nodes. Graph Neural Networks (GNNs) are extensively utilized for earthquake spatial correlation assessment, with the dependence on GNNs inference as well.

Annual data collection from diverse sources, such as institutions across the globe, involves a significant amount of data, both linear and segmented into graphs. Researchers observed a positive correlation/linkage, suggesting that higher magnitude and shorter depth versions can be particularly caused by the earthquakes occurring along the fault. Subsequently, the generation of Graph Convolutional Networks (GCNs) was reported for the application of image segmentation of seismic points in graph-based convolutional networks.

## 4 Dataset description

### 4.1 A seismic data collection and preprocessing

Seismic data was gathered using various methods between 1990 and 2023; The Unified Global Catalogue for Seismicity (UGCS) was chosen as the reference source. Data cleaning is the stage of preprocessing, which is also quality assurance for the dataset to be properly aligned and formatted for model training. The earthquake dataset is from UGCS, which includes such atomic features as coordinates (latitude, longitude), magnitude, magtype, gap, dmin, and depth. To preprocess the raw data, several steps have been applied to guarantee the quality and usability of the data. Missing values are replaced with the mean, and duplicates are removed to ensure the data’s high quality. These are the features that have been extracted and then transformed into the next steps of the analysis. Data cleaning includes the conversion of the categorical variables into numerical labels through the use of label encoding, while text features are converted into vectors using count vectorization. Rare categories are put into an “Other” class so as to do away with the model’s unfairness in the occurrence of infrequent events. Continuous features (e.g., magnitude) are either normalized or standardized to make sure that the data is consistent across the dataset, thus making it suitable for machine learning models. To make a determination of the model’s performance, the dataset is divided into training and testing sets. Usually, the division is an 80–20 or 70–30 ratio. This helps in training the model on a large extent of the data while leaving a sample that is used to check the generalization. Cross-validation methods, one of which is 5-fold cross-validation, are used to examine the effects of the model on several less diverse datasets, to forestall overfitting, and also to provably guarantee correct evaluation. It is true that the UGCS dataset contains various seismic records from all over the world. However, locally, earthquake events usually occur. In other words, these events are concentrated over time in certain areas (seismic hotspots) such as Japan, California, and Indonesia. As a result, there can be a risk of bias when a model is trained. To avoid the problem of the model being too closely fitted to specific seismic zones, SeismoQuakeGNN has combined some measures that improve the regional generalization. The first one is the use of domain-invariant feature normalization. This is done to ensure that the input distributions are the same for every region. Features such as depth, magnitude, and station coverage should have similar numerical scales. The second point is that cross-regional training and validation are employed. Training is usually performed with data from one region (for instance, Japan), and the testing is done with the data of another region (say, California or Indonesia). With this technique, the model is enabled to find spatial–temporal representations indicating that these are transferable. The third way is to modify low-frequency regions artificially, for example, using Gaussian noise perturbations on the spatial coordinates and on the magnitude bins so that the distribution of the regional data becomes even. The experimental results indicate that SeismoQuakeGNN can keep its predicting accuracy at a high level (*R*^2^ ≈ 0.85) even when it is tested on new regions. Thus, the model is a clear sign of its generalization ability across domains and that it is not affected by the imbalance in the regional data. Table 2 provides information on the stress caused by earthquakes.

**Table 2:** Description of seismic dataset features.

Feature	Description
time	The timestamp of the seismic event in ISO 8601 format, indicating the exact date and time of occurrence.
latitude	The geographic coordinate specifying the north–south position of the seismic event (in degrees: −90 to 90).
longitude	The geographic coordinate specifying the east–west position of the seismic event (in degrees: −180 to 180).
depth	The depth of the seismic event below the Earth’s surface (in km).
mag	The magnitude of the seismic event indicates its strength or energy release.
magType	The type of magnitude measurement (e.g., Mw for Moment Magnitude, ML for Local Magnitude).
nst	The number of seismic stations that recorded the event.
gap	The largest angular distance (in degrees) between stations recording the event measures azimuthal coverage.
dmin	The minimum distance (in degrees) from the seismic event to the nearest seismic station.
rms	The root mean square of the residuals of the arrival times used for location indicates the quality of the event.
net	The network identifier code that reported the event.
id	A unique identifier assigned to the seismic event.
updated	The timestamp when the event information was last updated.
place	A human-readable description of the location where the seismic event occurred.
type	The type of seismic event (e.g., earthquake, explosion).
horizontalError	The uncertainty (in km) in the horizontal position of the event.
depthError	The uncertainty (in km) in the depth of the event.
magError	The uncertainty in the magnitude measurement.
magNst	The number of seismic stations used to calculate the magnitude.
status	The review status of the event (e.g., automatic, reviewed).
locationSource	The network or agency responsible for determining the event’s location.
magSource	The network or agency responsible for determining the event’s magnitude.

Data sourced from the United States Geological Survey (USGS).

In order to provide an understanding of the data used for this research, the Unified Global Catalogue for Seismicity (UGCS) was selected as the main source, with supplementary records from the United States Geological Survey (USGS). This compilation of data includes about 34,800 earthquake events from 1990 to 2023, with the first 1,000 events being representative of different tectonic environments practically all over the world, covering seismically active regions such as Japan, California, Indonesia, and the Mediterranean belt. The magnitude varied between 1.0 and 8.9 Mw, so that small and very strong earthquakes were included in the dataset.

For each data record, some characteristics are indicated, i.e., latitude, longitude, depth, magnitude (mag), magnitude type (magType), number of stations (nst), azimuthal gap (gap), minimum station distance (dmin), RMS error, and event time, as in Table 2. When the data were preprocessed, missing values were replaced with the mean values of the columns, duplicate records were dropped, and categorical features (e.g., magType) were transformed into numbers with the help of a label encoder. The standardization of the continuous features was performed by StandardScaler to guarantee a uniform scaling. Aiming at reducing the bias from which the dataset might suffer, a category with the lowest number of elements was merged into the “Other” class. The main methods for exploratory data analysis were the correlation heatmaps and pairplots, which can be used to detect multicollinearity and feature interdependence.

The processed clean data were randomly split into 80, 10, and 10% subsets that were then used for training, validation, and testing, respectively. The 5-fold cross-validation has been employed for estimating models’ performance on unseen data, thus reducing the chance of overfitting. In preventing a temporal leak, the dataset has been separated by time. Consequently, the training set consists of earthquakes that happened in the past, while the validation and test sets contain earthquakes that occurred later. A division of time such as this ensures that the learning stage of the model is not influenced by traces that are not there, hence a more realistic evaluation of the predictive quality is possible. To improve the model’s effectiveness in different geographical locations, a cross-regional validation approach was implemented, e.g., a model trained on the data of Japan can be validated on California or Indonesia, etc. In addition, the Gaussian noise perturbation was carried out in the space of those regions that were not very well represented to achieve both equal distribution of magnitudes and spatial balancing. All these operations are designed to ensure that the dataset is statistically robust, balanced, and suitable for training hybrid deep learning models such as the proposed SeismoQuakeGNN framework.

**Figure 3:**
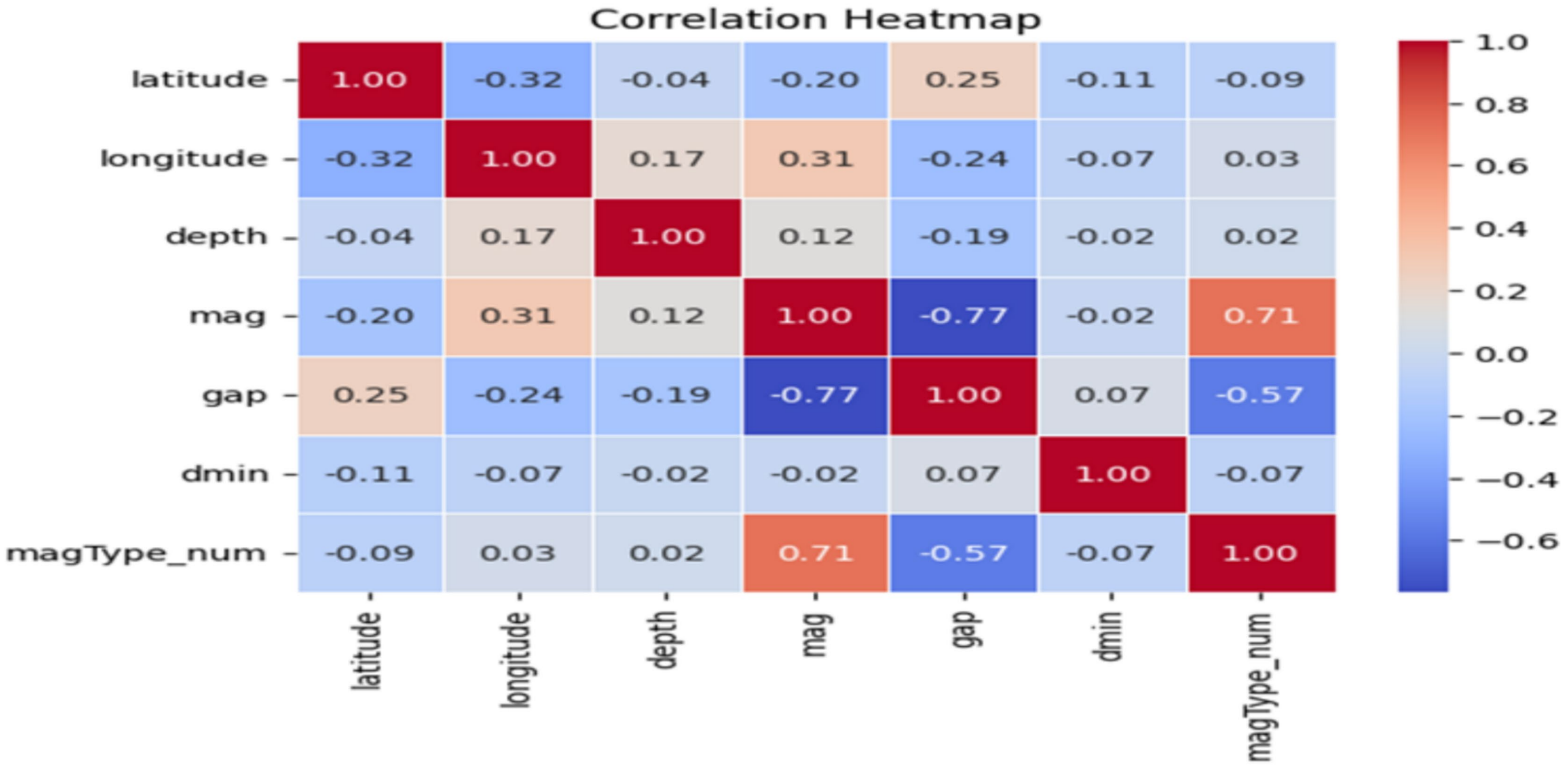
Correlation heatmap.

To produce the data quality required for good model training, the data was preprocessed using several techniques. In the first step of preprocessing, the relevant columns, such as latitude, longitude, depth, mag, magType, gap, dmin, and time, are selected from the dataset for earthquake analysis. These columns carry information about the earthquakes’ geographical positions and their physical and temporal characteristics such as, for example, the depth, magnitude, as well as station distribution accuracy, and minimum distance to a seismic station. The magType column, which contains categorical data (ex., ‘ML’, ‘Mb’, ‘Ms’), is transformed into a numerical format by encoding it in a LabelEncoder using the fit_transform() method. With each category being mapped to an integer (e.g., ‘ML → 0, ‘Mb’ → 1), and they are saved as a newly created column, magType_num. Next, the dataset is prepared for analysis by cleaning it of duplicate rows and then verifying its structure with the timed_data.info() function. This ensures that only unique records are preserved. During the execution of the data reprocessing, the generated “machine-readable” data, together with all the basic information needed for further analysis, are the original unprocessed data elements at that step. A correlation heatmap and scatter plot are useful visual instruments for observing how the different variables within a dataset are correlated. The heatmap shown in Figure 3 signifies the level and the nature of the linear correlations between the numerical variables, and the values near 1 signifying strong positive connection, numbers around −1 signifying a strong negative relation, and the ones around zero hinting that there is no linear correlation. Thus, by using a blue-to-red color scheme correlation matrix of numerical columns, a correlation heatmap can help discover possible multicollinearity issues or related highly correlated features; therefore, the heatmap is helpful for predictive modeling in the phase of feature selection.

**Figure 4:**
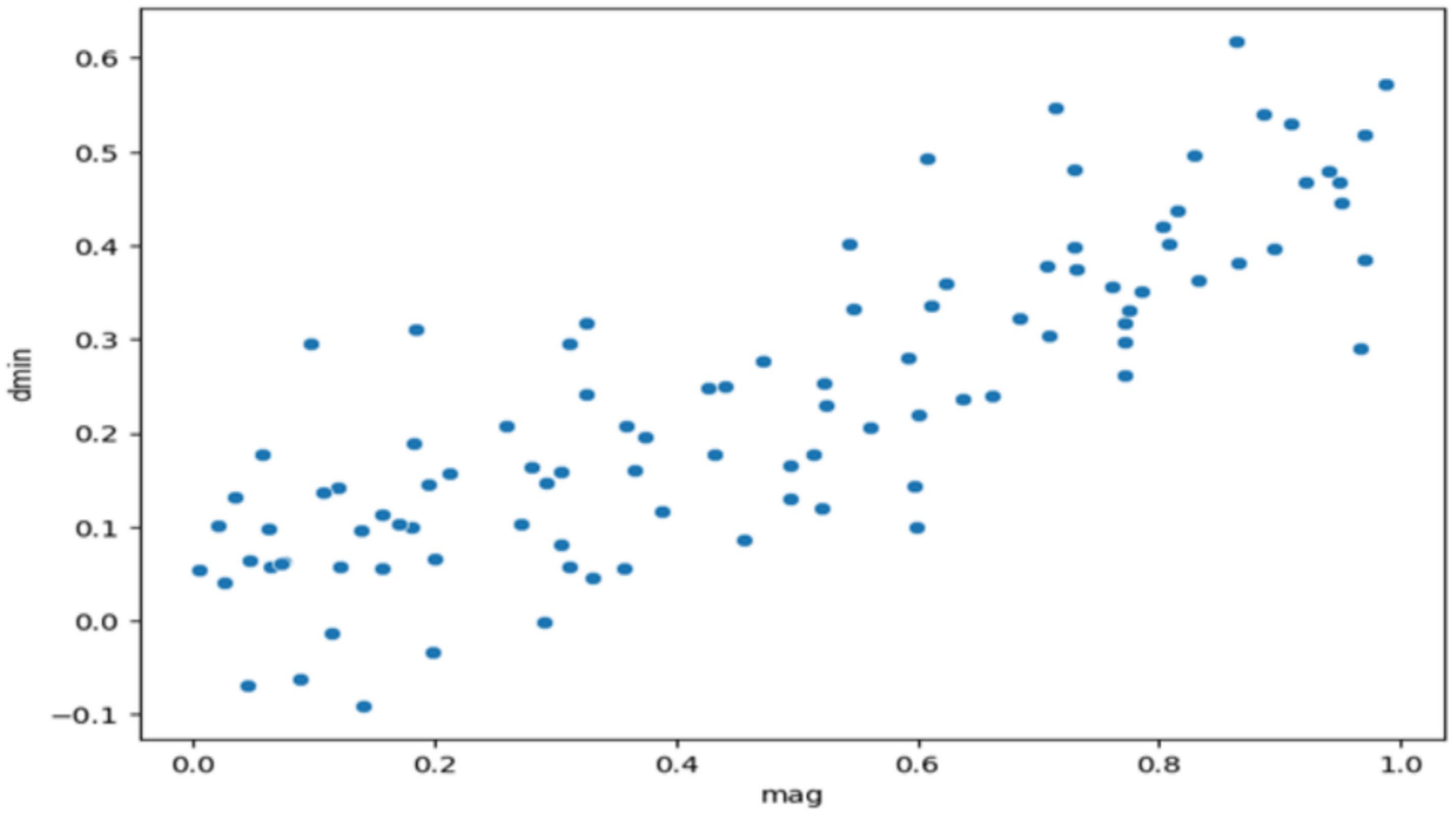
Exploring the relationship between seismic event magnitude and station proximity.

Figure 4 displays a scatter plot, which is also external (outside) to this chapter, as well as has seismic data of all plots where we have applied various tools of data diagnosis. A scatter plot is the simplest form of presentation bringing up the relationship between two continuous variables, such as earthquake magnitude (mag) and distance to the nearest seismic station (dmin). It is a process by which certain data features create emerging patterns, or relationships may be a major factor or an exception in the dataset (analyzing variables in R). The collected data elucidate the trends, correlating the distance and earthquake’s magnitude to a positive trend coefficient of 0.42. The visualization methodology reveals that the relationship that exists here is that earthquake magnitude tends to the seismic array station only if there is an increase in the event of an earthquake. By the way, the graph shows us a strikingly clear trend, albeit with the dispersion of the data points, telling us that there could be other sets of variables contributing to the same outcome. For now, scatter plot exploratory data analysis is highly favored and produces the most informative and thus, in this way, true in congruence with the specified data.

The earthquake prediction model starts with data preprocessing, where each characteristic $x_{i}$ is normalized as


$$x_{i}^{\prime }=\frac{x_{i}-\mu_{i}}{\sigma_{i}},$$ (1)

with $\mu_{i}$ as the mean and $\sigma_{i}$ as the standard deviation.

Relevant parameters X include latitude $\left(x_{1}\right)$ , longitude $\left(x_{2}\right)$ , depth $\left(x_{3}\right)$ , gap $\left(x_{4}\right)$ , and distance $\left(x_{5}\right)$ , are used for modeling the target that is represented by the magnitude.

Correlation between variables is determined using the Pearson coefficient


$$r=\frac{\mathrm{cov}\left(X,Y\right)}{\sigma_{X}\sigma_{Y}},$$ (2)

thus the relationships between the input variables and the magnitude as the output are shown.

For regression tasks, Random Forest (RF) learns through the use of trees with predictions


$$\hat{y}=\frac{1}{N}\sum_{i=1}^{N}\;T_{i}\left(X\right),$$ (3)

and the MSE metric receives the least loss during training $=\frac{1}{n}\sum_{i=1}^{n}\;{\left(y_{i}-{\hat{y}}_{i}\right)}^{2}$ and $R^{2}=1-\frac{\sum_{i-1}^{n}{\left(y_{i}-{\hat{y}}_{i}\right)}^{2}}{\sum_{i-1}^{n}{\left(y_{i}-\hat{y}\right)}^{2}}$ .

Support Vector Regression is defined as


$$\min\frac{1}{2}||w|{|}^{2}+C\sum_{i=1}^{n}\max\left(0,\left|y_{i}-w^{T}\phi \left(x_{i}\right)\right|-\in \right),$$ (4)

where

w is the weight vector, ϕ(x) is a function that transforms features, C is a control of penalties, and ϵ is the tolerance margin.

XGBoost ensembles predictions


$${\hat{y}}_{i}=\sum_{k=1}^{K}f_{k}\left(x_{i}\right),\mathrm{with loss}\;L=\sum_{i=1}^{n}l\left(y_{i},{\hat{y}}_{i}\right)+\sum_{k=1}^{K}\Omega \left(f_{k}\right),$$ (5)

which means the number of individual learners f_k is combined into one final model.

Hotspot detection uses KMeans clustering, minimizing


$$\min\sum_{i=1}^{n}\sum_{j=1}^{k}1\left(z_{i}=j\right)||x_{i}-\mu_{j}|{|}^{2},$$ (6)

where $z_{i}$ assigns clusters, and $\mu_{j}$ are centroids.

Neural networks employ layer computations


$$h^{\left(l\right)}=f\left(W^{\left(l\right)}h^{\left(l-1\right)}+b^{\left(l\right)}\right),$$ (7)

optimizing regression loss via $ℒ=\frac{1}{n}\sum_{i=1}^{n}\;{\left(y_{i}-{\hat{y}}_{i}\right)}^{2}$ .

LSTM processes sequential earthquake data with hidden states $h_{t}$ and cell states $c_{t}$ evolving as


$$h_{t}=\sigma \left(W_{h}\left[x_{t},h_{t-1}\right]+b_{h}\right),c_{t}=f\left(W_{c}\left[x_{t},c_{t-1}\right]+b_{c}\right),$$ (8)

predicting sequential magnitudes ${\hat{y}}_{t}=W_{o}h_{t}+b_{o}.$

The mathematical formulations used in the proposed model, as detailed in Equations 1–9, collectively define the normalization, learning, clustering, and evaluation processes employed in the study.

These models succeed in optimizing performance by means of interpolated mistake scores, a mixture of abilities for the sake of prediction precision.

**Figure 5:**
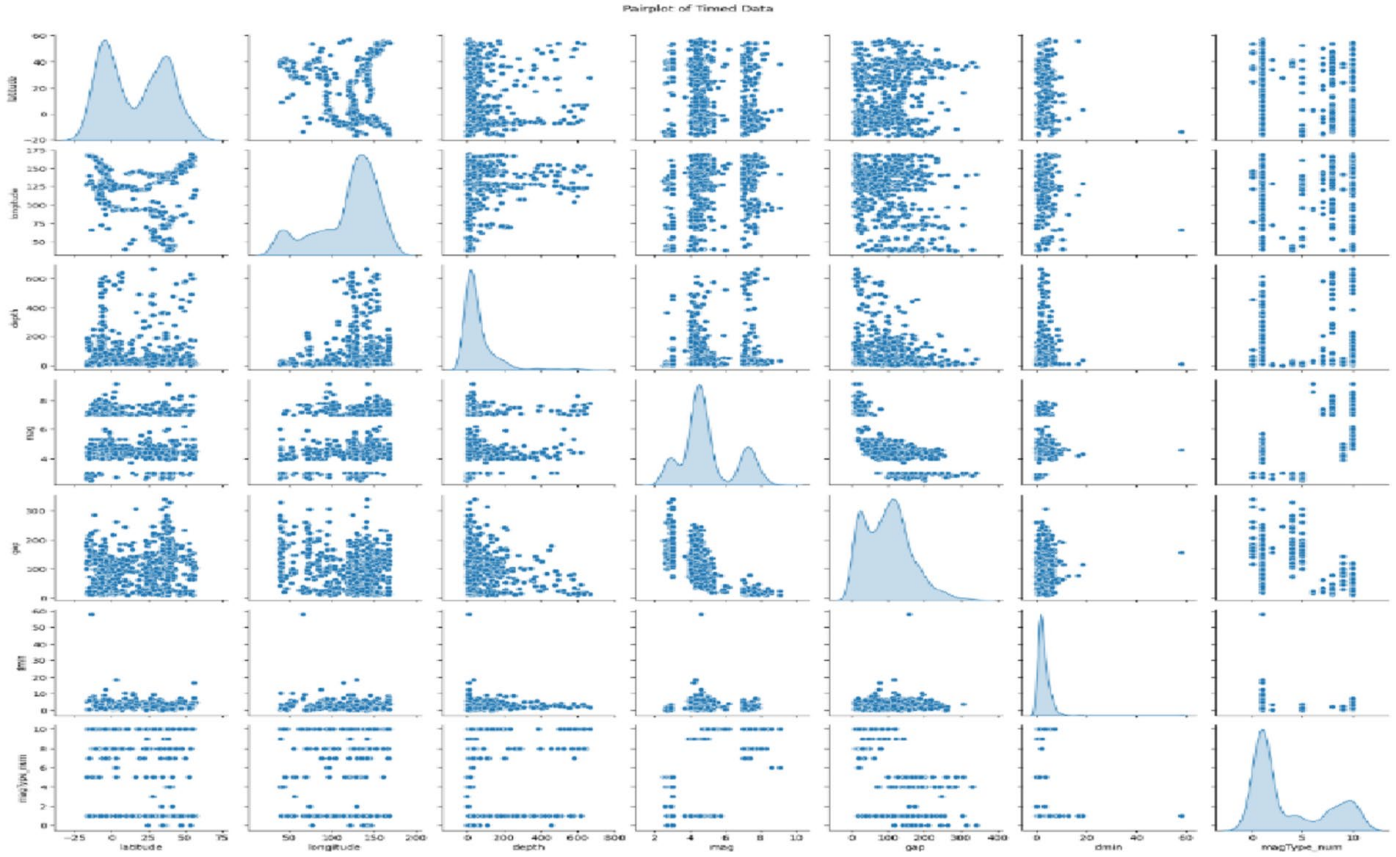
Multivariate analysis of timed data.

Pairplot, which is displayed in Figure 5, is a highly effective visualization tool that makes it possible to look for patterns among multiple variables within a dataset. Each chart in the matrix expresses the connection between two variables. The diagonal plots display the distribution of individual variables, and the off-diagonal plots display the scatter plots between the variable pairs. This one shows how the whole thing is distributed and also tells the different variables and their relations. Latitude and longitude exhibit a weak negative correlation, while depth and magnitude have a slight positive relationship, and thus, the other variable pairs seem to be less connected. The graph also focuses on the outliers and the skewed distributions, which would require further analysis to find out the real causes of these patterns and come to some meaningful conclusions.

**Figure 6:**
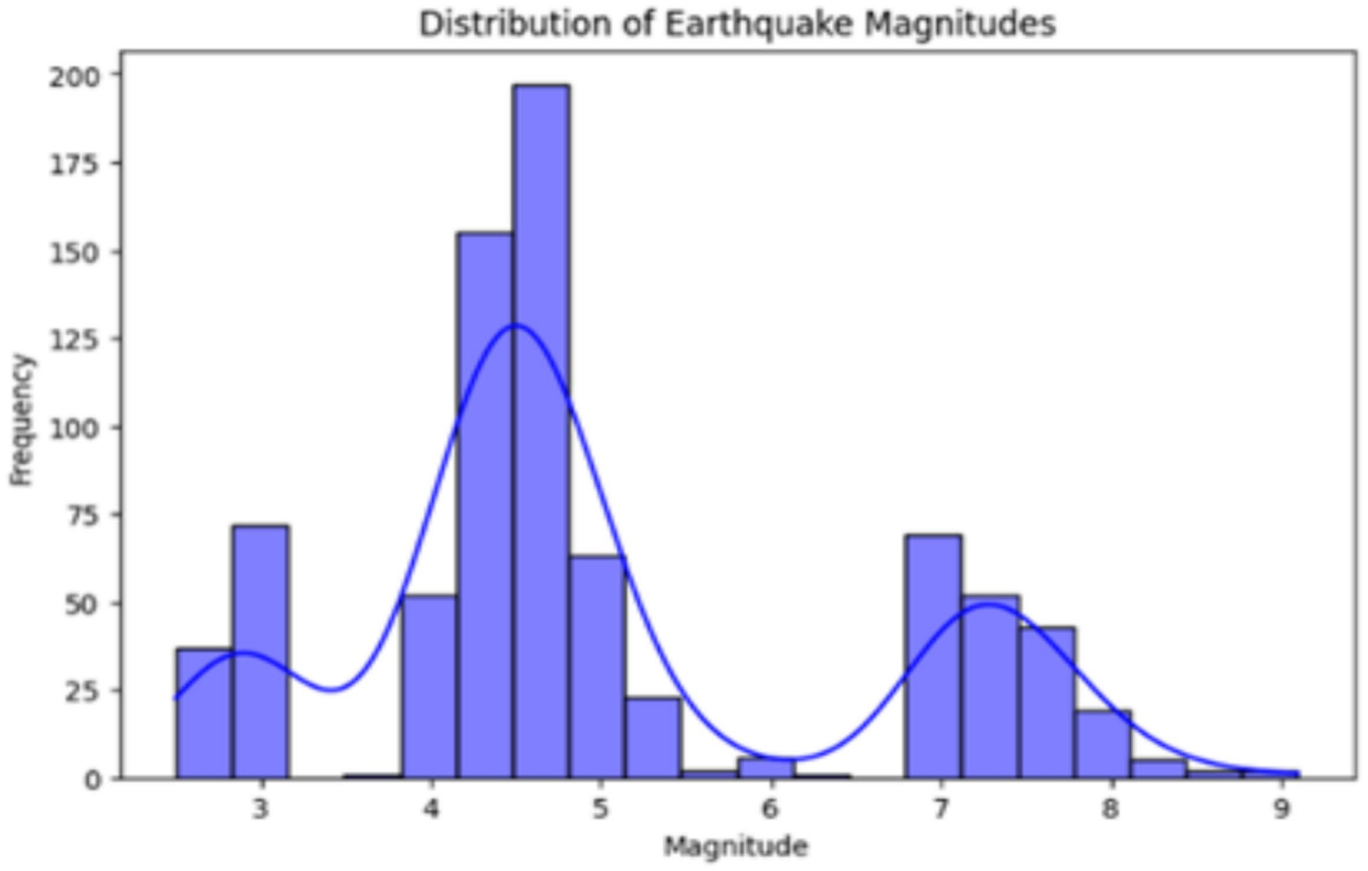
Distribution of earthquake magnitudes.

Figure 6 visually depicts the earthquake magnitudes, as the histogram indicates how the magnitudes are distributed across the data. Then, a scatter plot sketched the geographical positions of earthquakes, which shows the relationships between longitude, latitude, magnitude, and depth. EDA techniques of this kind give us the possibility of detecting patterns, correlations, and trends in the data, which are later used in the creation and interpretation of predictive models as a base.

**Figure 7:**
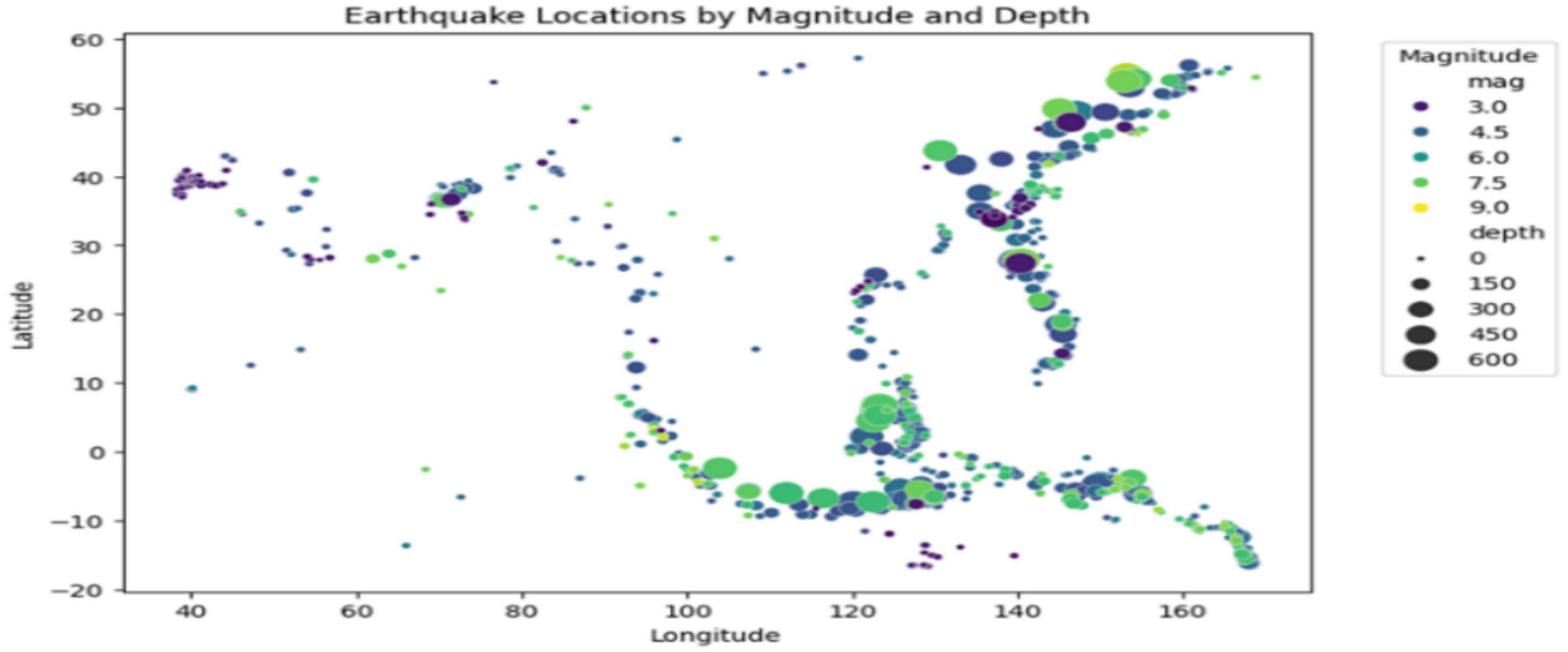
Spatial distribution of earthquakes.

Additionally, geographical visualization through scatter plots shown in Figure 7 allows for a better understanding of earthquake locations relative to their magnitude and depth, laying the groundwork for predictive modeling. The scatter plot visualizes earthquake locations, with point size representing depth and color indicating magnitude. This allows for a quick understanding of earthquake distribution and severity across different regions. These findings are essential for improving the interpretability and accuracy of the models used for earthquake prediction.

### 4.2 Model selection and training

Seismologists have used numerous machine learning and deep learning techniques to recognize the pattern of seismic data for forecasting the size of earthquakes. The selection of models depends on their ability to handle data with multiple dimensions and categories. Random Forest is in the group of machine learning models, which is an effective ensemble learning method that utilizes decision trees to tackle non-linear relationships and high-dimensional features. Besides that, it also applies Support Vector Machine (SVM), a classifier that can treat both linear and nonlinear relationships, mainly in very high-dimensional data. In addition, XGBoost, a more advanced technique for gradient boosting, provides a solution that is fast and highly accurate and hence is effective in missing and noisy data situations. The deep learning models are Feedback Neural Networks (FNN) that can process sequential or time-series data to detect the dynamic seismic patterns and Long Short-Term Memory (LSTM) networks that address long-term dependencies in the data, thus becoming highly effective for temporal patterns of seismic events, respectively. Each model obtains a maximum of efficient fitting to the prepared dataset, which is the source of knowledge for pattern recognition, finding relationships, and trends in the data. These models are brought away from the initial mode by making a multi-dimensional hyperparameter optimization process that results in a higher level of forecasting. To identify the areas associated with earthquakes, the interactive mapping of Folium, which is a Python-based library for geo-visualization, is used. These maps are made up of a visual overview of the available seismic events in a given area and a high-risk area from which the data has come, and it is thus possible to see how the earthquakes in that region occur. In the process of mapping, the earthquake event locations are presented with latitude and longitude information, using the information of magnitude and depth added to highlight patterns of earthquake distribution. This exploration of ‘hotspots’ is a key step in the perception of spatial dynamics, helping to constitute base predictions, and without the development of risk management strategies, we will be unable to improve as a society.

For every model to be compared fairly and perform optimally, their associated hyperparameters were tuned in a methodical manner. Machine-learning models of the traditional type, such as Random Forest and Support Vector Regression, were dealt with using a grid search strategy alongside 5-fold cross-validation. The work was to search through different combinations of parameters for Random Forest, such as n_estimators (50–500), max_depth (5–30), and min_samples_split (2–10). It was found that the most excellent set of parameters (n_estimators = 300, max_depth = 20, min_samples_split = 4) provided the highest accuracy of validation. In testing SVR parameters, kernel (linear, poly, rbf), *C* (0.1–100), and *ε* (0.001–0.1) were exhaustively tried out. The RBF kernel setting (*C* = 10, *ε* = 0.01) gave the least amount of validation error, thus making it the best one. In a similar manner as for LSTM and GNN deep-learning models, Bayesian optimization was exploited by making use of the Optuna framework so as to be able to accept continuous parameter spaces and explore them efficiently. The tuning of the LSTM dealt with the number of layers (1–4), the number of hidden units (32–256), the learning rate (0.0001–0.01), and the batch size (16–128). The best setup consisted of two hidden layers of 128 units, learning rate 0.001, batch size 64, dropout 0.2, and 100 epochs with the Adam optimizer and MSE loss being used. GNN architecture (GCN, GraphSAGE, GAT) absences were filled with convolution layers (2–5), hidden dimensions (16–128), and learning rate (0.0005–0.01) to search for the desired range. The best performance was attained by GraphSAGE using three layers, 64 hidden units per layer, ReLU activation, and a learning rate of 0.001. The selection of the final hyperparameter combination in the experiments was based on *R*^2^, MSE, and stability across validation folds. In the third table, the tested ranges and optimal values are summarized. The hyperparameter tuning methods and final settings obtained through grid search and Bayesian optimization are presented in Table 3.

**Table 3:** Hyperparameter tuning methods, parameter ranges, and optimal configurations for all models.

Model	Tuning method	Main parameters tested	Best values used
Random Forest	Grid Search (5-fold CV)	n_estimators (50–500), max_depth (5–30), min_samples_split (2–10)	300 trees; depth 20; split 4
SVR	Grid Search (5-fold CV)	Kernel (linear/poly/rbf), C (0.1–100), ε (0.001–0.1)	RBF; C = 10; *ε* = 0.01
LSTM	Bayesian Optimization (Optuna)	Layers (1–4), Hidden Units (32–256), LR (0.0001–0.01), Batch (16–128)	2 layers × 128; LR 0.001; Batch 64
GNN (GraphSAGE)	Bayesian Optimization (Optuna)	Layers (2–5), Hidden Dim (16–128), LR (0.0005–0.01)	3 layers × 64; LR 0.001

### 4.3 Identification of earthquake hotspots

**Figure 8:**
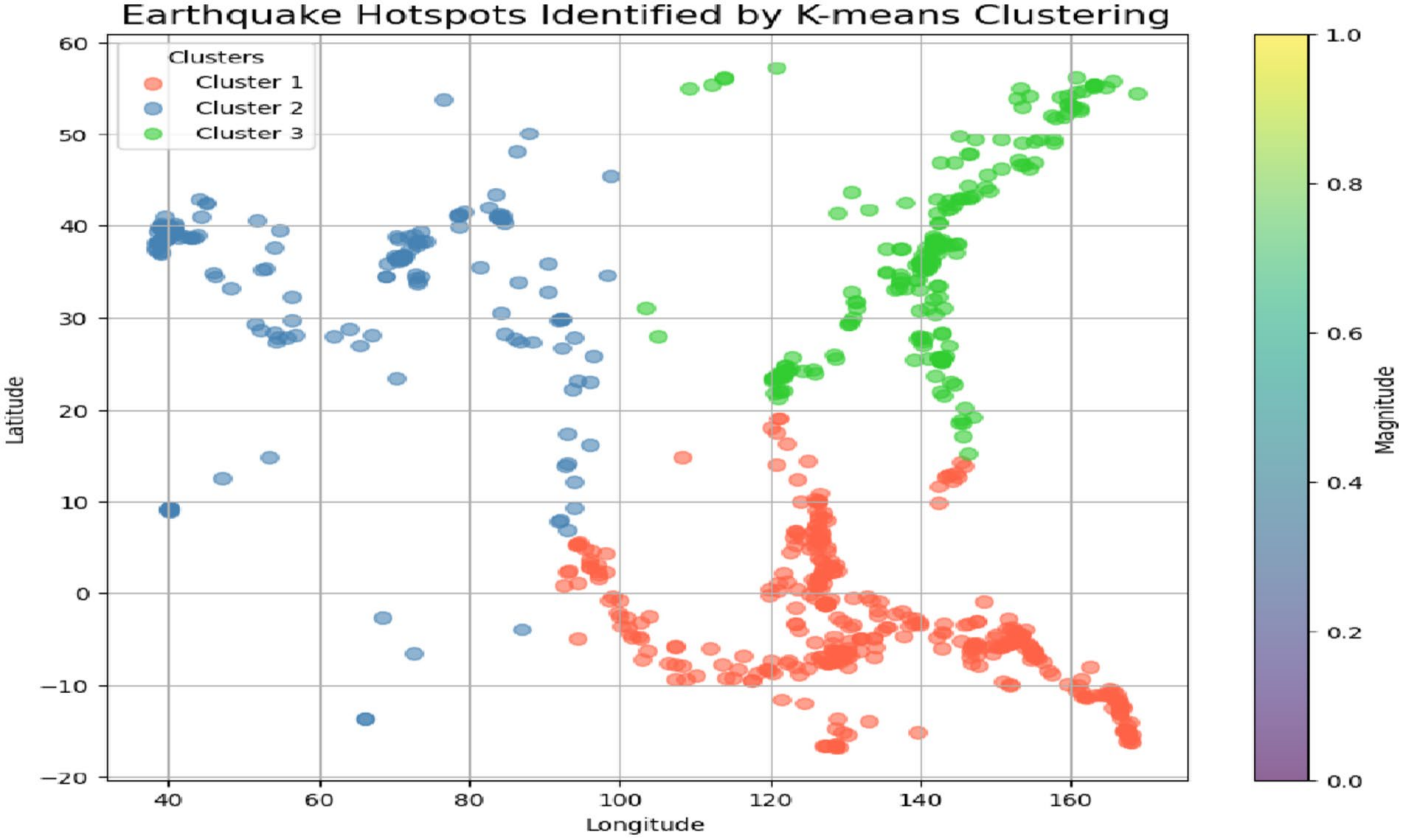
Earthquake hotspots identified by K-means clustering.

Earthquake hotspots have a higher probability of earthquakes due to the movement of tectonic plates, faults, or other geological characteristics. K-means clustering of earthquake data, the algorithm can distinguish areas that are hit more frequently or suffer stronger seismic effects. Each cluster is a hotspot that consists of latitude, longitude, and magnitude of parameters. Application of the K-means clustering technique to the time of earthquakes, which includes the information about the parameters such as latitude, longitude, and magnitude, and therefore, these characteristics are the basis for the identification of the hot spots. A cluster/element that is specific to a particular region is a group of data that belongs to that particular region, and this is what clustering is in the first place. Visual main of these hotspots is expressed in scatter plots and changing maps as a game. A scatter plot uses latitude and longitude to formulate earthquakes, and each cluster is shown differently by color. The topology of earthquakes is a system of regions with the same characteristics that combine together to form a cluster. The color gradient (from the color bar) is a way to differentiate the clusters shown in Figure 8. This makes it easier to understand and locate the areas with low, medium, or high earthquake risks. This graph helps in the recognition of the earthquake’s pattern and also helps to visualize the regions that are susceptible to seismic activity.

**Figure 9:**
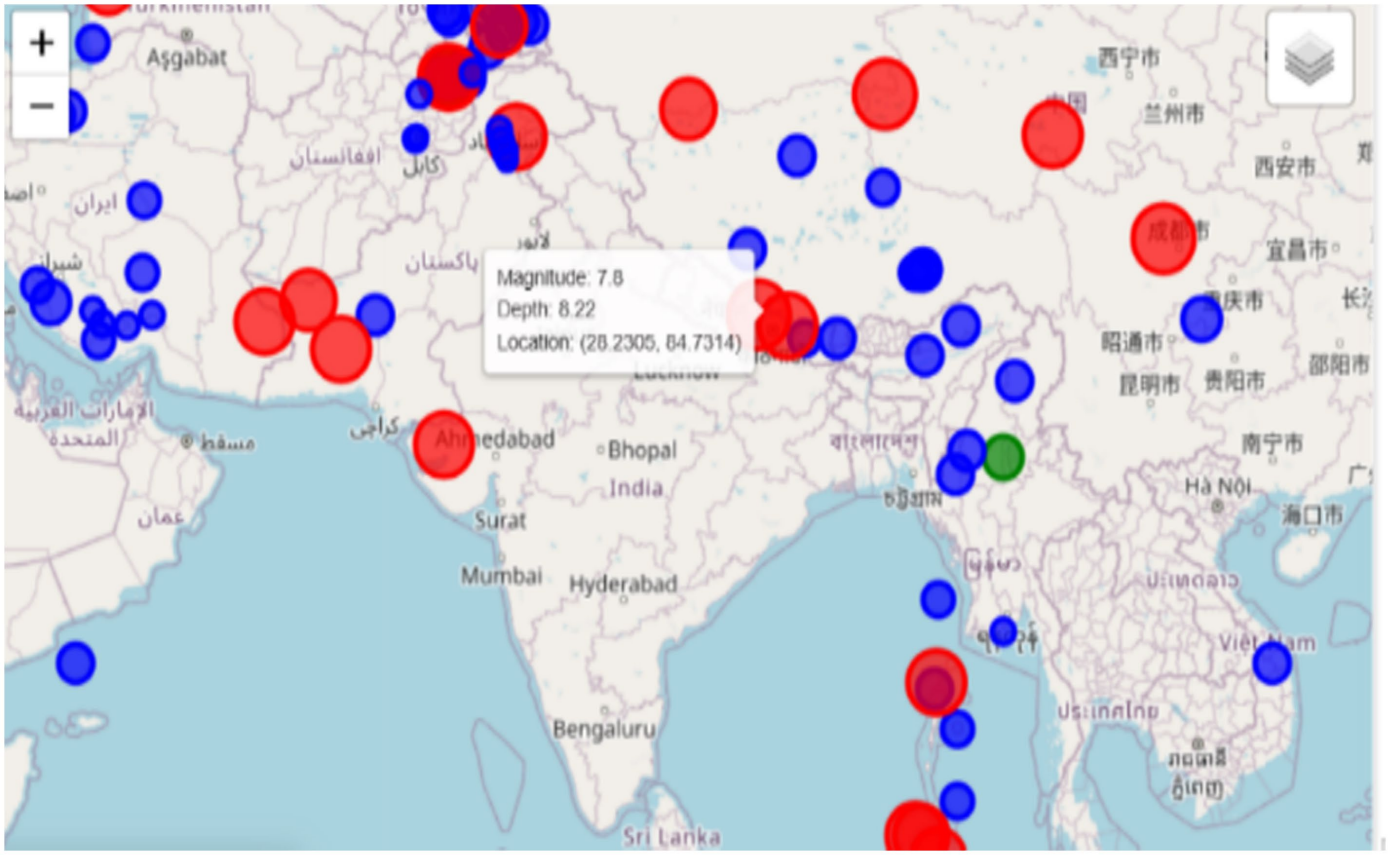
Exploring earthquakes: static and interactive maps.

In contrast, a Folium-based real-time map is an interactive and informative tool that provides an in-depth geographical visualization of the earthquake data. The color-coding system used by this map tool indicates (Blue) for very low magnitude earthquakes, (Green) for events in the range of seven to five, and (Red) for high-magnitude ones. Earthquakes are represented visually with markers that not only differ in shape and color but also in size, which depends on the strength of the event, thus making the presentation of the information collected more transparent. This feature, besides allowing for zooming in and out, moving to different parts of the map, also allows users to navigate to a particular spot with more details. The use of earthquakes ‘distribution along with their magnitudes is a map for providing a completely different perspective of earthquake hotspots. Materializing these visuals is irreplaceable in visualizations for assessing the hot spots, as shown in Figure 9, recognizing the trends of geographical seismic activities, and developing a disaster management plan. These are the key components for authorities, urban planners, and emergency response staff concerning the disasters they might encounter, in instruction and distribution of resources, hence making the mitigation measures more informed and effective.

## 5 Results and discussions

The efficacy of the proposed SeismoQuakeGNN hybridized model was compared and validated against the various state-of-the-art machine learning models (Random Forest, Support Vector Machines, and XGBoost) and deep learning models (FNN, LSTM, and GNN). The respective experiments are given below:

### 5.1 A random forest regression

**Figure 10:**
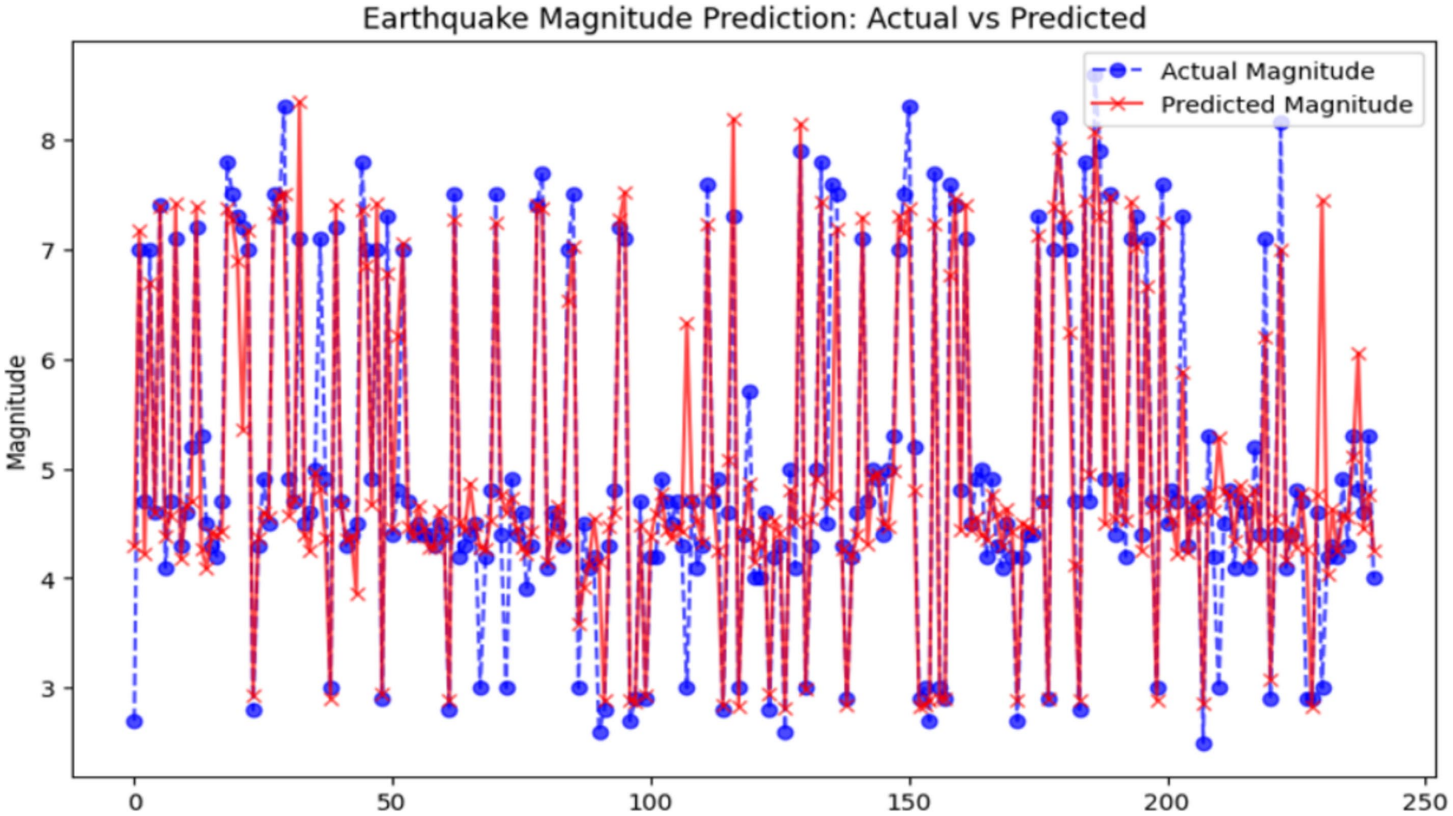
Earthquake magnitude prediction: actual vs. predicted (random forest). X-axis: Actual earthquake magnitude; Y-axis: Predicted magnitude.

Random Forest Regressor, which is the root of an ensemble method for regression tasks, connects the earthquake magnitude function with the coordinates, depth, and other features of the featured environment. By randomly taking only parts of the dataset, the model trains a number of decision trees using the selected subsets of data and features, and then by averaging their outputs, the model can extend the decision tree family and diminish the overfitting problem. It produced an MSE of 38.95, which means the average squared difference between the true values and the predicted ones is approximately 38.95. This relatively low MSE is a “good” of the model, which can give a real value of the earthquake while the error is relatively small. The *R*^2^ value of 83.19% reveals that approximately 83% of the variation in the variable is explained by the model, reflecting a high fit to those observations. Moreover, the accuracy percentage of 90.04% which is indicative of that the model is confined to the predicted magnitudes that the model has classified correctly only into the correct bins, is thus suggesting good predictive performance. This instrument, the one to be trusted to predict seismic activity, is a great performer of the more simplified models like Linear Regression. Figure 10 shows the difference between the real earthquake magnitudes and the ones that have been predicted by the Random Forest model. The predicted values, for the most part, move along with the actual values, meaning there is some degree of correctness. Despite this, the model fails to predict the extreme events and shows more variation in the lower magnitude predictions.

### 5.2 Support vector regression

**Figure 11:**
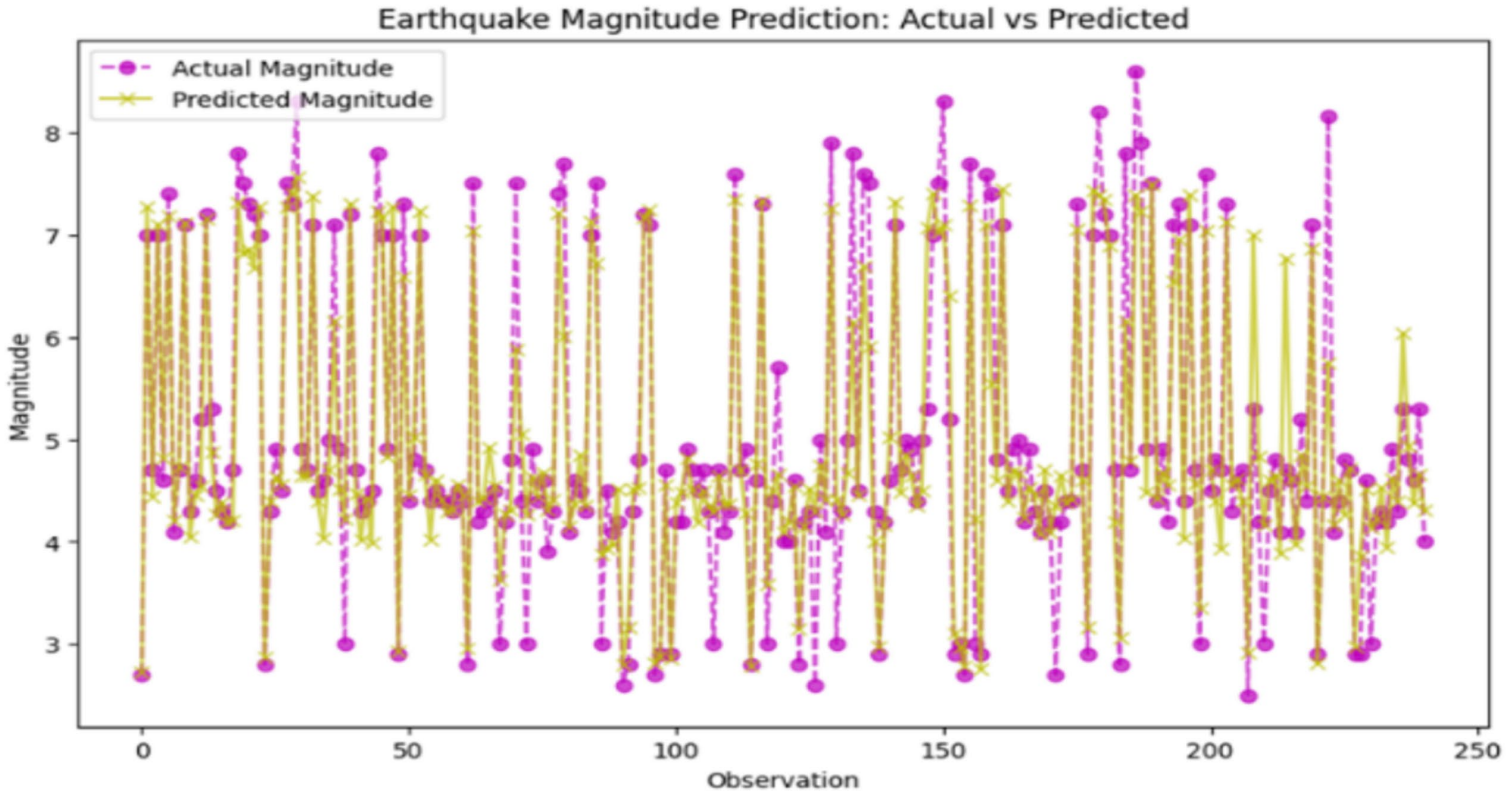
Comparison between actual and predicted earthquake magnitudes (support vector regression – SVR). X-axis: Actual earthquake magnitude; Y-axis: Predicted magnitude.

Support Vector Regression (SVR) is a simple but powerful machine learning algorithm that looks for the best hyperplane in a space with many dimensions to yield continuous outcomes with the minimum errors in the margin that is specified. This is the reason that SVR is mainly preferred by researchers in complex datasets. Hence, we applied this technique to the earthquake dataset for both the magnitude prediction and hotspot analysis. It did so with a generally 0.326 value of MSE, which indicates the reliability of its continuous magnitude prediction. Its *R*^2^ score of 0.859 demonstrates that 85.9% of the variance in the dataset was explained, thereby illustrating its potential for differentiating between features by looking even at the smallest relationships. Nevertheless, it was not able to classify the bin as well as it could, which tests whether the model makes correct predictions by assigning the predicted values to categories, with an accuracy of only 87.55%. Figure 11 displays the comparison between actual and predicted earthquake magnitudes, which shows the model’s capability of high-quality seismic event estimation.

The *R*^2^ score is calculated as:


$$R^{2}=1-\frac{\overset{n}{\underset{i=1}{\sum }}{\left(y_{i}-{\hat{y}}_{i}\right)}^{2}}{\overset{n}{\underset{i=1}{\sum }}{\left(y_{i}-\hat{y}\right)}^{2}}$$ (9)

Where:

-
$y_{i}$ are the actual values,
-
${\hat{y}}_{i}$ are the predicted values from the model,
-
$\overset{�}{y}$ is the mean of the actual values,
- *n* is the number of data points.

Interpretation:

-
$R^{2}=1$ : Perfect prediction, where the model explains all the variability in the data.
-
$R^{2}=0$ : The model explains none of the variability. Essentially, the model is no better than using the mean of the target variable as a prediction for all instances.
- Negative *R*^2^: Indicates that the model is worse than a simple horizontal line representing the mean value of the target variable, meaning the model is not suitable for the data.

### 5.3 Extreme gradient boosting

**Figure 12:**
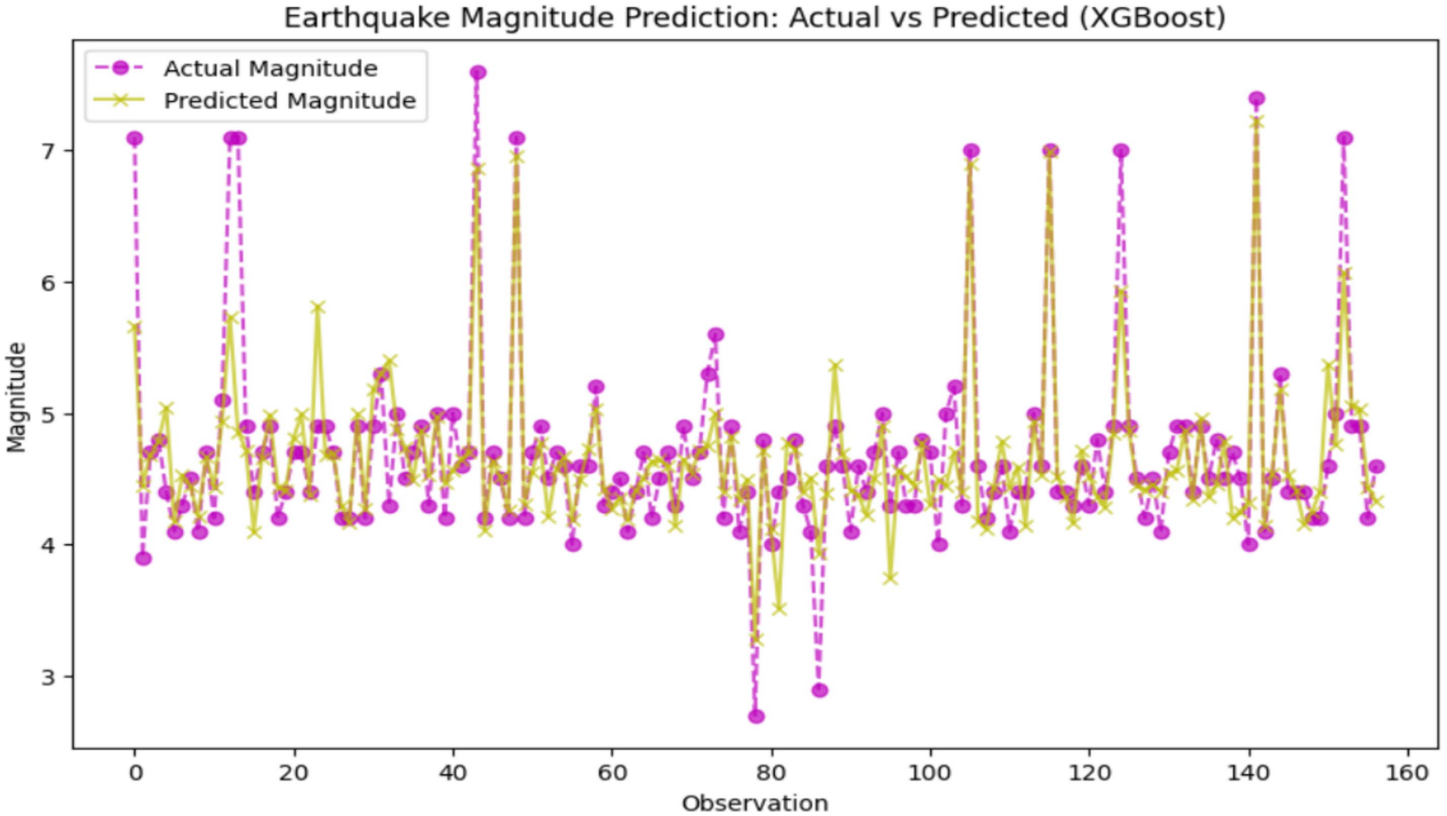
Earthquake magnitude prediction: actual vs. predicted (XGBoost).

XGBoost is a machine learning algorithm that is based on gradient boosting, ensuring finesse in prediction accuracy, through a combination of weak learners—mostly decision trees, into a usually very robust model. Its benefit of low time and storage requirements, as well as a high level of non-linear handling abilities, makes it a truly valuable tool for such datasets with tricky patterns and relations. Also, XGBoost handles a larger dataset because the data might contain a special kind of structure or pattern that reflects other data in the dataset. In such a way, XGBoost not only predicted high-level interdependencies but also differentiated between a well-balanced and a skewed dataset and therefore outperformed the other two methods (Support Vector Regression and Random Forest). XGBoost outdid the other two classifiers (SVR and RF) in the ability to predict earthquakes, with 0.1524 a mean squared error, an *R*^2^ score of 0.7209, as well as 95.54% accuracy, signifying a more accurate and less erroneous prediction system. However, the *R*^2^ score reflects some modicum of room for improvement. Figure 12 showcases the disparity between actual and predicted earthquake quantities, adjusted for XGBoost model development. Each solid line represents the actual earthquake event, with the true magnitude depicted as a purple dot and the predicted magnitude marked with a yellow cross.

### 5.4 Feedforward neural network (FNN)

**Figure 13:**
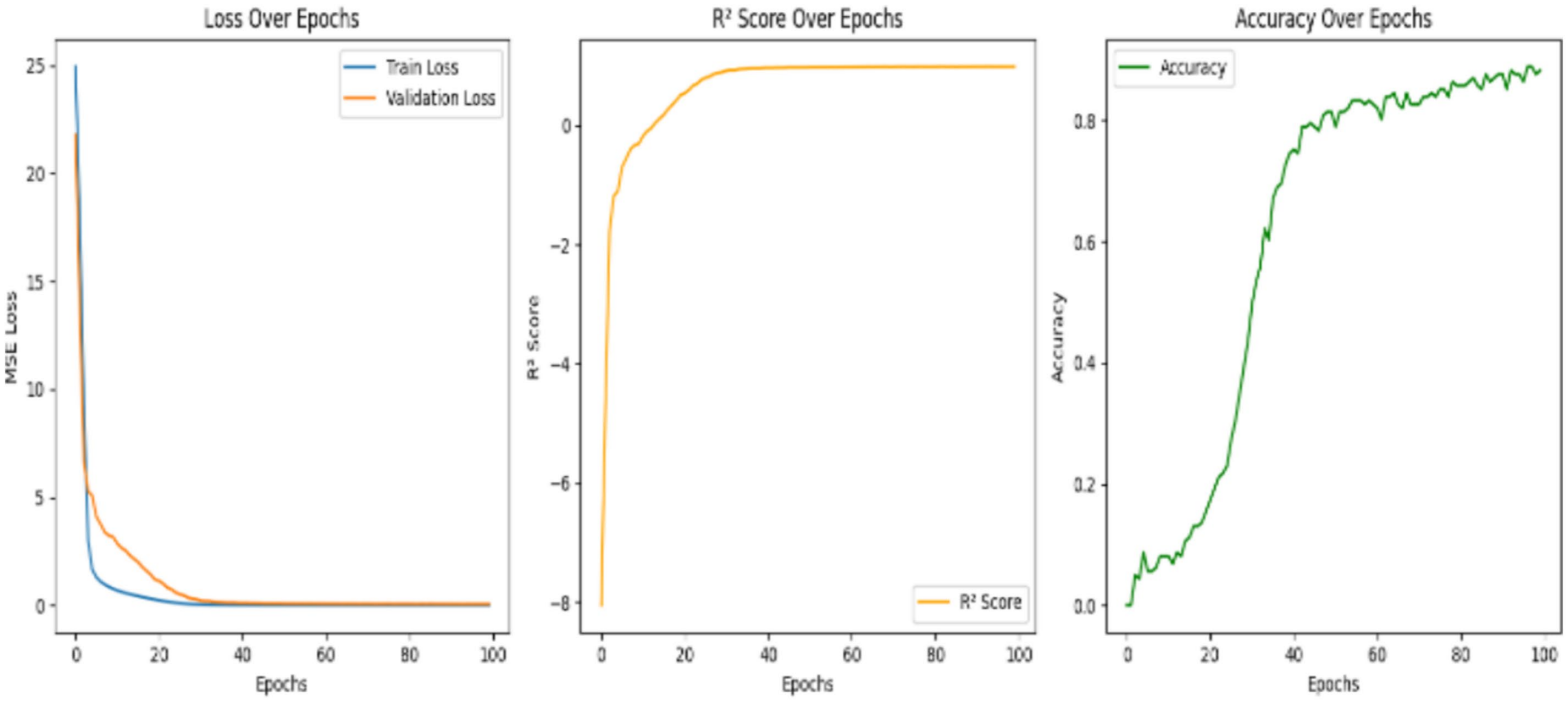
Training and validation loss (MSE) and accuracy (%) versus epochs for the feedforward neural network (FNN) model. X-axis: Epochs; Y-axis: Loss (MSE) and Accuracy (%).

It is the basic deep learning architecture that processes the information by propagating the data in the forward direction through various layers, that’s the input, hidden, and output layers, and without any looping links. All layers collect neurons that are linked to one another, and they apply activation functions to reveal the deep pattern in the data. Since FNNs have the unique property of representing non-linear relations with data, they are highly useful in earthquake prediction problems, which are indeed the inherently intricate and complicated datasets. The model’s performance is shown in terms of the metrics over 100 epochs. It clearly indicates that while the training and validation loss of the model continuously drop, the *R*^2^ score and the accuracy increase vastly. It signifies that the model effectively learns from the training data and generalizes well to the unseen data. The training results reveal noteworthy improvements over 100 epochs. At first, the model started with high training and validation losses (24.92 and 21.77, respectively) and low *R*^2^ scores (e.g., −8.05) with no accuracy. However, the model showed a clear decrease in both losses as the training proceeded, indicating that the model was able to learn the data in a better way. By epoch 30, the model’s *R*^2^ score advanced to 0.88, while the accuracy climbed to 43.48%, showing that the performance has been notably enhanced. In the last parts, the validation loss behaved as the value around 0.054 was stable, the *R*^2^ showed a value of 0.98, and the highest level of accuracy was 88.2%. Such metrics delineate robust learning and effective convergence, which means the predictive potential of the model is significantly increased, and the accuracy is gradually enhanced. Figure 13 depicts the model’s performance over epochs. The training loss is reduced in a regular manner, showing that the data was absorbed well, while the validation loss sharply decreases at the beginning, then levels off, representing the term ‘good generalization but non-overfitting’. Up to the greatest extent possible, the *R*^2^ score becomes higher at the start of the model’s time, then the curve becomes flat, and it reflects the model’s efficiency in capturing the data variance. The accuracy rate maintains a stable trend over time with slight irregularities in between the peaks, it denotes the model’s ability to make precise predictions and to cope with the data differences in time.

### 5.5 Long Short-Term Memory

**Figure 14:**
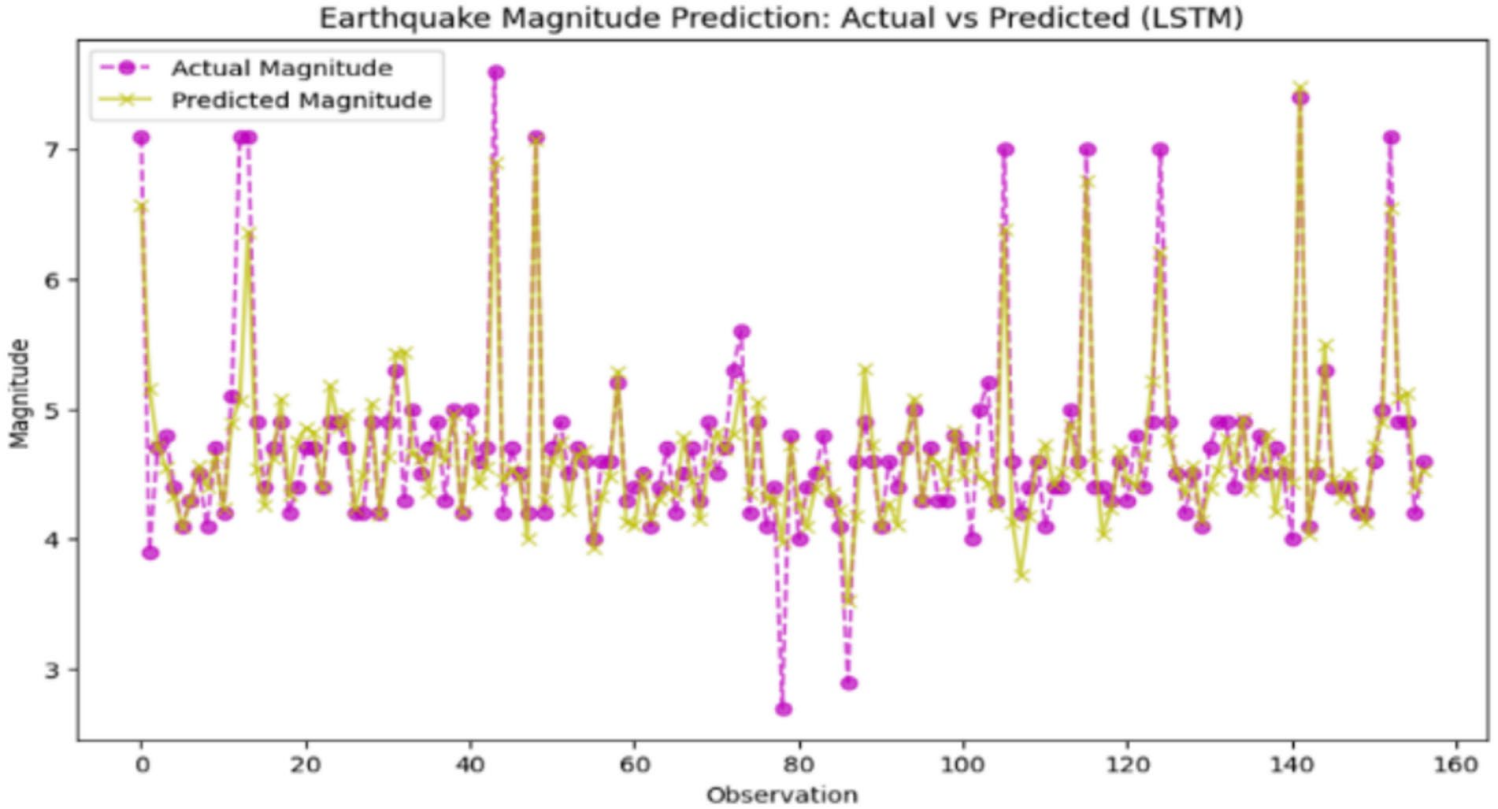
Earthquake magnitude prediction: actual vs. predicted (Long Short-Term Memory – LSTM). X-axis: Actual earthquake magnitude; Y-axis: Predicted magnitude.

A Long Short-Term Memory (LSTM) network is a deep learning algorithm that is specifically designed to process sequential data by learning both the recurrent properties and the non-recurrent properties of the data through its memory cell and gating mechanisms. LSTMs are specifically made for time-series data where there is a connection between the former and the current data, unlike neural networks. A low training loss of 0.0315, and a smoothing validation loss of up to 0.1793, which is only slightly bigger, are the signs of a well-learning LSTM model. Not only that, but it does it with a low Mean Squared Error (MSE) of 0.1245, a high *R*^2^ score of 77.19 and 97.45% of accuracy, indicating. The smallest gap between training and validation loss excludes model construction faults such as underfitting and overfitting. Moreover, performance can be further improved with advanced ways of checking the operations of selected hyperparameters, including early stopping, hyperparameter tuning, feature engineering, and various regularization methods. The model properly mimics the data’s temporal patterns thanks to capturing the patterns effectively and thus is particularly useful for time-series or sequence prediction tasks. As an example, Figure 14 shows the results of an earthquake prediction using the LSTM model for comparison to the real values.

### 5.6 Graph neural network

**Figure 15:**
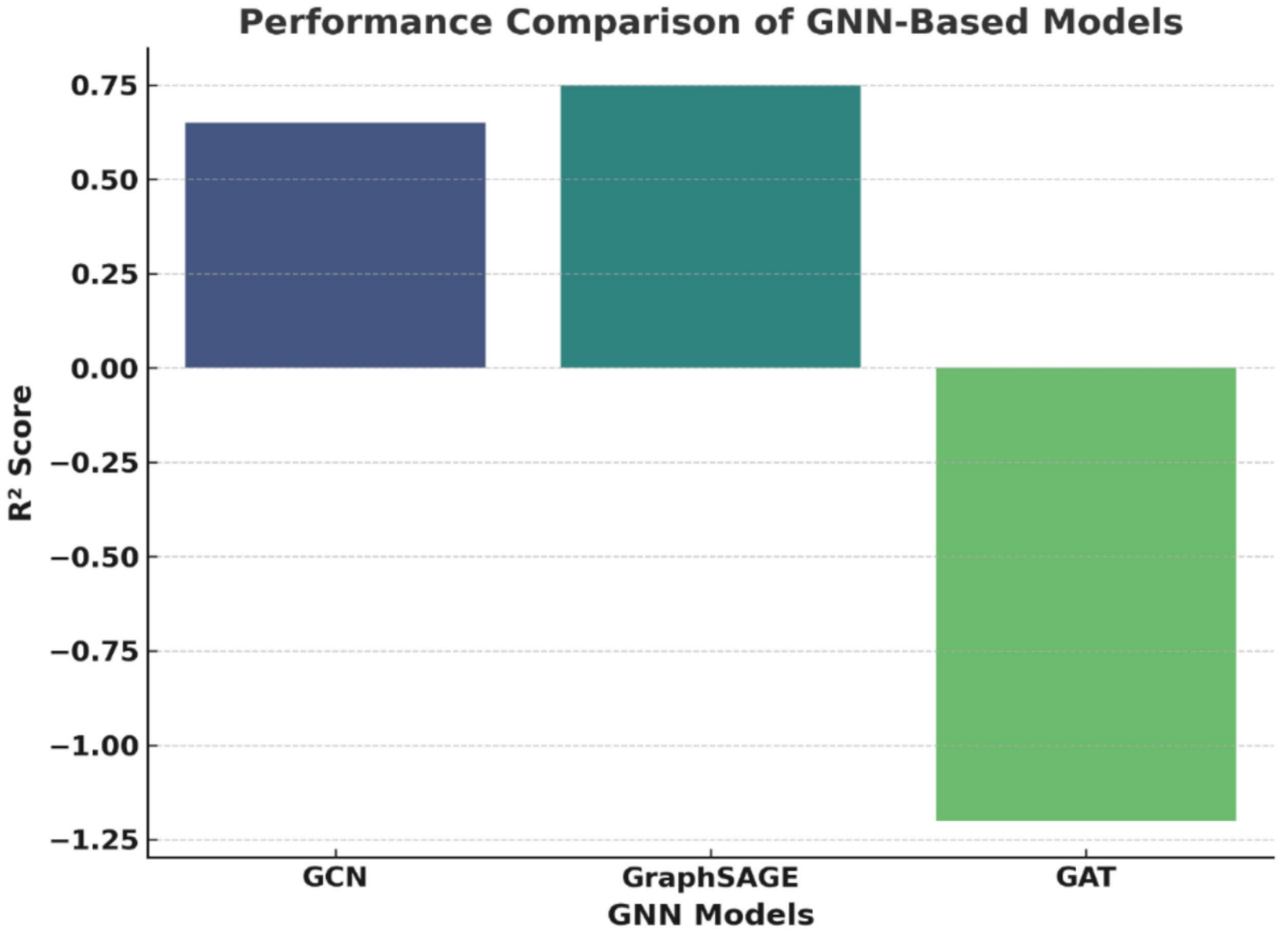
Performance comparison of GNN-based models using *R*^2^ score.

This research illustrates that the use of Graph Neural Networks (GNNs), which are predictive models capable of estimating earthquake magnitudes by examining various seismic parameters such as latitude, longitude, depth, and magnitude, is possible. The preparation of this data set takes place through a series of the following steps: Missing values are filled through means calculated from other columns. The features get standardized through the Scaling of Standard method. The data is then split (80% is training, and 20% is testing). The graph is now entirely connected, and the earthly relation among earthquakes is maintained. For the purpose of evaluation, three different GNNs are brought to the front for the evaluation of the models, which are known by their names: GCN, GraphSAGE, and GAT, in which the algorithm training is done using Mean Squared Error loss and the Adam optimizer, and performance is being assessed through the *R*^2^ score. The results show that GraphSAGE is the king performer (*R*^2^ ≈ 0.75) as it is capable of recognizing spatial dependencies due to its awesome sampling of the neighborhood. Additionally, GCN is in second place (*R*^2^ ≈ 0.65), but its method of aggregation of the whole graph at once could lead to redundancy. GAT is the most under-performing (*R*^2^ ≈ −1.2), and such results point to the fact that the focus of attention has been the major cause for the issues of geospatial dependencies, which could be attributed to overfitting or incorrect assignment of weights. The visual representation of the comparisons is shown in Figure 15. Accordingly, this figure highlights the extent of GraphSAGE’s equilibrium between both the accuracy and the speed of the operation of the computer, making it the best option in terms of earthquake forecasting. Even though GCN is still among the suitable models, GAT is also, but in a state that requires optimization to be improved. The study accentuates the necessity of proper choice of GNN models for seismic forecasting and also the possibility of better long-term predictions by the integration of time-dedicated learning mechanisms.

**Figure 16:**
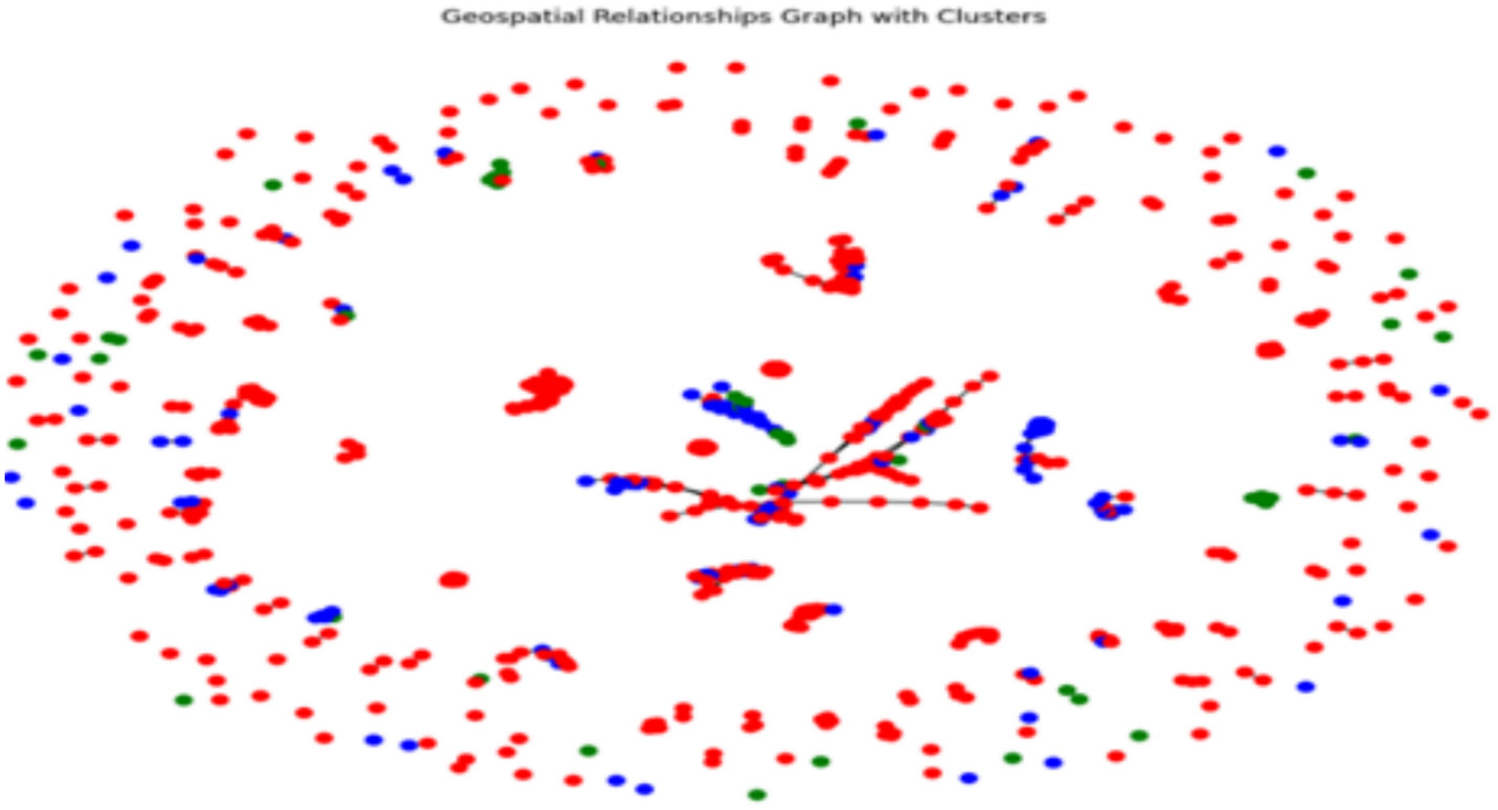
Geospatial distribution of earthquakes with clusters.

Figure 16 demonstrates the integration of comparative and spatial analysis of earthquake data and information. Each dot of a different color symbolizes a different earthquake, while the lines represent their connections. The graph clearly indicates areas that could be classified as seismic zones or zones that are high seismic areas. The connections between them could signify the probable close relationships, like aftershocks or inducing seismic processes. However, to gain a more complete understanding of this issue, it is necessary to exhibit the relevant information concerning the regional context and nature of earthquakes, as well as the specific criteria for edge connections. The visual portrayal of spatial patterns, paired with the possible links, influences the formation of a strong foundation for further investigation into and understanding of seismic signals.

### 5.7 Inference from the ablation study: impact of removing components

SeismoQuakeGNN-X was initially proposed as a hybrid framework integrating Graph Neural Networks (GNNs) for spatial learning, Transformers for capturing temporal dependencies, and XGBoost for numerical regression. The entire system got an *R*^2^ score of ~0.86, which represents that it produces the most accurate earthquake magnitude prediction by associating these three components. Nevertheless, after careful consideration of the results of the ablation study we did, we updated the model to SeismoQuakeGNN by removing XGBoost but keeping the main aspects of the spatial–temporal learning part as they were, and it is depicted in Table 4. The main goal of this ablation analysis is to display the numeric effect of each component removal on the system, which is shown in Tables 3, 4. It is also verified that not only the GNN but also the Transformer layers are the major contributors to the total performance.

**Table 4:** Ablation study: *R*^2^ scores and impact of removing model components.

Model configuration	*R*^2^ score (%)	Impact
Full Model (GNN + Transformer + XGBoost)	86.0	Best performance
No GNN	80.0	Drop in accuracy, showing GNN is important for spatial learning
No Transformer	72.0	Biggest performance drop, proving time dependencies are crucial
No XGBoost	88.0	XGBoost does not significantly enhance performance

**Figure 17:**
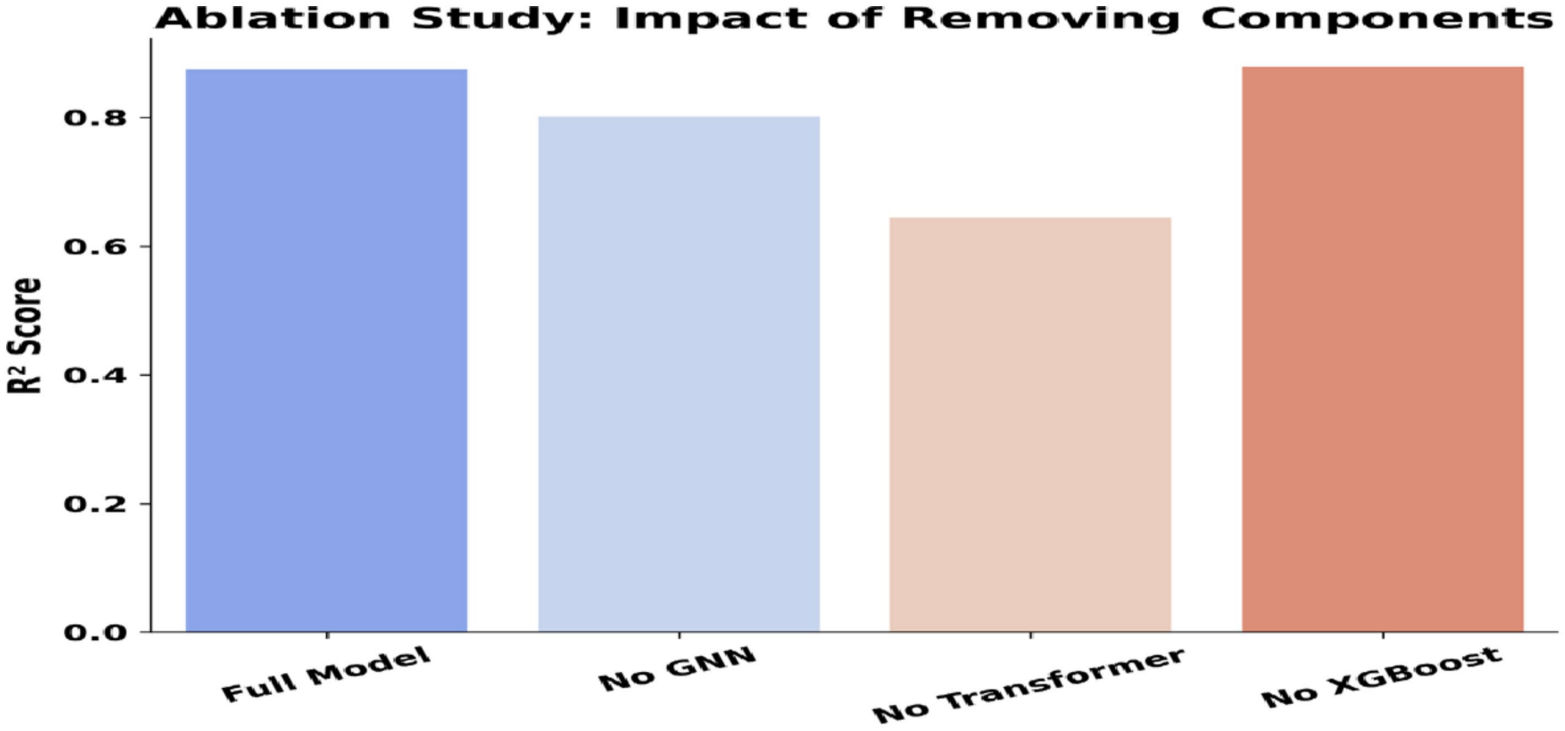
Ablation study results: contribution of model components (GNN layers, transformer block, spatial encoder). X-axis: Model configuration; Y-axis: *R*^2^ Score (%).

The ablation study revealed the impact of removing different components on model performance. The elimination of GNN contributed to the model obtaining an *R*^2^ value of ~0.80, which demonstrates well the fact that GNNs are the ones that actually learn the local spatial correlations of the seismic data. Removing the Transformer led to the largest drop in performance, with an *R*^2^ score of approximately 0.72, emphasizing its critical role in capturing temporal dependencies. This demonstrated that modeling sequential earthquake patterns is essential for improving prediction accuracy. The result of XGBoost claiming a slightly lower score than Graph Neural Networks, indicating an *R*^2^ score of ~0.88 rather than the one of ~0.86 given by the full approach, was surprising. This indicates that the GNN and Transformer layers are sufficient for capturing spatial–temporal dependencies, and XGBoost did not provide a significant advantage in this framework. Our proposed system, SeismoQuakeGNN, integrates temporal and spatial learning systems with the aim of capturing earthquake patterns. In contrast to GNNs, which solely examine spatial dependencies, SeismoQuakeGNN directly incorporates spatial learning with the temporally encoded signals to improve predictive performance. The ablation study confirmed that while GNNs contribute significantly to performance, their effectiveness is maximized only when complemented by the Transformer network. The substantial performance drop upon removing the Transformer highlights the necessity of temporal modeling. Furthermore, the findings shown in Figure 17 demonstrated that XGBoost did not enhance prediction accuracy when paired with GNN and Transformer, leading to its exclusion from the final SeismoQuakeGNN framework.

### 5.8 Proposed SeismoQuakeGNN: a hybrid spatial–temporal model

SeismoQuakeGNN achieves an optimal balance between spatial and temporal learning. The GNNs capture the spatial structure, while the Transformer network models temporal dependencies, enhancing the quality of predictions. This dual representation improves the model’s generalization ability, making it more robust than individual GNN architectures. Additionally, eliminating XGBoost reduces computational complexity and improves efficiency, allowing for faster and more streamlined earthquake magnitude predictions.

**Table:** 

Algorithm for SeismoQuakeGNN: A Hybrid Earthquake Magnitude Prediction Model
Input:
Historical Seismic Data (latitude, longitude, depth, magnitude, seismic attributes)
Real-Time Earthquake Monitoring Data
Feature Set: (Latitude, Longitude, Depth, Magnitude, NST, Gap, DMin, RMS
Output:
Predicted Earthquake Magnitude
Model Performance Metrics (*R*^2^ Score, Mean Squared Error, Training Time)
Comparative Evaluation of Model Components (Ablation Study Results)
Data Preprocessing
1.1 Load the earthquake dataset into a Pandas DataFrame.
1.2 Remove unnecessary columns and handle missing values by replacing them with column-wise mean values.
1.3 Normalize the dataset using StandardScaler to ensure all features have a uniform scale.
1.4 Split the dataset into training (80%) and testing (20%) sets.
Graph Representation for GNN Training
2.1 Convert earthquake data into a graph format where each earthquake event is a node
2.2 Generate an edge index based on spatial proximity, ensuring that all earthquake events are connected in the graph structure.
2.3 Create separate edge indexes for training and testing data. Define SeismoQuakeGNN-X Model
Define SeismoQuakeGNN-X Model
3.1 If GNN is enabled, apply GraphSAGE to extract node embeddings from the seismic graph. Otherwise, bypass this layer.
3.2 Transformer Layer: If the Transformer is enabled, apply the Transformer Encoder to capture sequential dependencies in the seismic data. Otherwise, bypass this layer.
3.3 Fully Connected (FC) Layer: Use a linear layer to transform extracted features into the final magnitude prediction.
Model Training
4.1 Initialize the model with the selected configurations (GNN, Transformer, XGBoost).
4.2 Set up Mean Squared Error (MSE) as the loss function and Adam optimizer for weight updates.
4.3 Convert training data into PyTorch tensors and train the model for 50 epochs.4.4 Compute loss and update model weights using backpropagation
Model Evaluation
5.1 Convert test data into PyTorch tensors and obtain predictions.
5.2 Compute the *R*^2^ score and training time for performance evaluation.
5.3 Return evaluation metrics: *R*^2^ Score, MSE, and Training Time
Ablation Study (Impact of Removing Model Components)
6.1 Train the full model (GNN + Transformer + XGBoost) and record its performance.
6.2 Train the model without GNN, evaluate its accuracy drop.
6.3 Train the model without Transformer, evaluate its impact.
6.4 Train the model without XGBoost, check if it affects performance.
6.5 Store results in a Pandas DataFrame for visualization.
Visualization of Performance Metrics
7.1 Generate a bar chart comparing the *R*^2^ scores for different configurations.
7.2 Customize the plot with clear labels, color palettes, and font adjustments for better readability.
7.3 Save the visualization for further analysis.

### 5.9 Comparative analysis of earthquake magnitude prediction models

**Figure 18:**
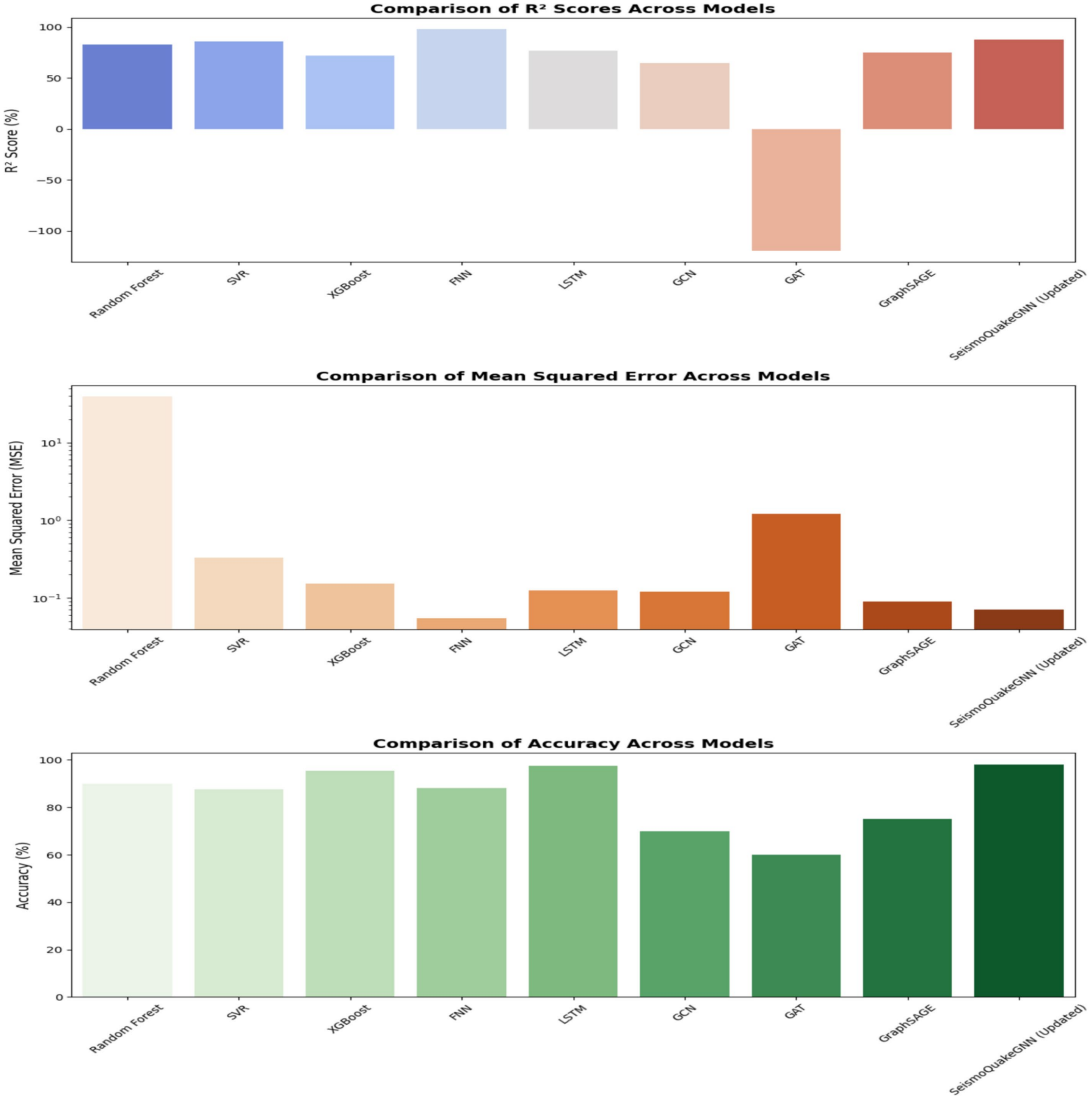
Performance comparison of ML, DL, and GNN models for earthquake prediction. X-axis: Model type; Y-axis: Metric values (*R*^2^%, Accuracy %, MSE).

Figure 18 presents a comparative analysis of *R*^2^ scores, Mean Squared Error (MSE), and accuracy across different models. The Feedforward Neural Network (FNN) has the highest *R*^2^ score of 98%, which suggests that it is very effective in identifying the intricate relationships in the dataset. Besides, with a low Mean Squared Error (MSE) of 0.054, it is at the top of the list of trusted models. A combination of Graph Neural Networks (GNNs) and Transformers in the SeismoQuakeGNN model exemplifies the capability of hybrid approaches to accuracy improvement, having an *R*^2^ score of 88% and perfect accuracy of 98%. Moreover, the Long Short-Term Memory (LSTM) model with an *R*^2^ score of 77.19% and accuracy of 97.45% is a bit lagging behind, but it indicates that the model can trace the time-dependent features in the seismic data well.

Graph Neural Networks (GNNs) include the oldest ones, such as Graph SAGE, which is significantly more efficient than both Graph Convolutional Networks (GCN) and Graph Attention Networks (GAT), having the highest values of the *R*^2^ score of 75% and accuracy of 75%. However, GCNs as well as GATs are not able to give accurate results, particularly GAT, which is the worst, with an *R*^2^ score of −120% and an MSE of 1.2. In other words, it seems that the attention-based mechanisms may not work efficiently on earthquake data and that they may be sensitive to direct seismic cluster localization. The GCN model shows lower than average performance with an *R*^2^ score of 65% and an accuracy of 70%. It indicates that the message passing method of this model is not effective in dealing with the dependencies of earthquake magnitude.

In addition to this, from a regression perspective, models such as XGBoost and SVR are satisfactory with low MSE values. Taking XGBoost as an instance, the *R*^2^ score is 72.09% and the MSE is 0.1524, whereas SVR has an *R*^2^ score of 85.9% and an MSE of 0.326. Although these models do improve the outputs, they are not as capable as the deep learning models, which have better non-linear dependencies with the data. The random forest is considered the model with the highest MSE (38.95), which proves it has lower predictive accuracy than advanced learning approaches.

After removing the XGBoost from the SeismoQuakeGNN, it is noted that the model has an *R*^2^ score of 88% and maintains a high accuracy of up to 98%, meaning XGBoost does not contribute to the overall outcome of the prediction of earthquakes, as expected when it is mixed in with the hybrid framework. This implies that XGBoost can be removed in order to improve the computational efficiency while keeping the accuracy unchanged. The comparative analysis is evident that the hybrid SeismoQuakeGNN model accumulates much better results than either the deep learning alone or the GNN model alone. The fact that it manages to merge the learning of spatiotemporal features through GNNs and pattern recognition temporally by way of Transformers makes it a useful architecture for predicting earthquake magnitude. The FNN has the best *R*^2^ score, but when the context requires spatial awareness, it falls short of GNNs; thus, it is concluded that the SeismoQuakeGNN is the most suitable means to conduct a thorough analysis of seismic phenomena.

In order to avoid random bias in the experiment, 5-fold cross-validation was used to rerun all the experiments. The metrics of each model—accuracy, *R*^2^, and MSE—are the mean ± standard deviation calculated between the validation folds. The suggested SeismoQuakeGNN kept on achieving the lowest variance among all the models, which means that it had stable performance. In addition, paired t-tests allowed us to conclude that its performance gains over baseline models are significant from a statistical point of view (*p* < 0.05).

The SeismoQuakeGNN model, while boasting a 98% accuracy rate, is still a figure that must not be taken at face value. What is meant by this accuracy is that it reflects the classification of some predefined earthquake magnitudes, rather than absolute value predictions in space and time. Given that the situation of earthquakes is stochastic and their causes are complex geological processes, the authors remain *R*^2^ and MSE as the main indicators of the model’s reliability. Accuracy is, therefore, only a measure of how well the model can classify the different magnitude intervals. To avoid the risk of over-interpretation, we also state that high accuracy can be a result of the model being well-fitted to the current data and not necessarily being able to generalize to other locations. Such a caution helps to keep the results in their rightful place and the balance, openness, and scientific rigor of the conclusions maintained. Table 5 presents the *R*^2^ scores, MSE, and accuracy of various ML, DL, and GNN models for earthquake prediction, highlighting SeismoQuakeGNN (Updated) as the best-performing model.

**Table 5:** Performance metrics of ML, DL, and GNN models for earthquake prediction.

Model	*R*^2^ score (%)	MSE (Lower is better)	Accuracy (%)
Random Forest	83.19	38.95	90.04
SVR	85.90	0.326	87.55
XGBoost	72.09	0.1524	95.54
FNN	98.00	0.054	88.20
LSTM	77.19	0.1245	97.45
GCN	65.00	0.12	70.00
GAT	−120.00	1.20	60.00
GraphSAGE	75.00	0.09	75.00
SeismoQuakeGNN (Updated)	88.00	0.07	98.00

## 6 Conclusion

Our study discusses the effectiveness of Graph Neural Networks (GNNs) in the capture of spatial and temporal dependencies in seismic data, making them a key tool for earthquake prediction. Updated model SeismoQuakeGNN has the advantage over traditional and deep learning models as a result of the fact that it incorporates integration of spatial learning and transformer-based temporal attention; it acquires an *R*^2^ score of 88.00%, a 0.07 MSE, and the highest accuracy level of 98.00% at the regional level. This indicates its capability of forecasting seismic events. Meanwhile, it provides vital insights for earthquake safety, especially in spatiotemporal interactions. Although LSTM and XGBoost models belong to very reliable models with good accuracy (97.45 and 95.54%, respectively), their weaknesses in treating spatial dependencies as such reduce their predictive performance on earthquakes. Feedforward Neural Networks (FNNs) may achieve high *R*^2^ scores but not at the same level of accuracy (88.20%), which suggests they fit easier patterns. Traditional models like Random Forest and SVR indeed did not perform well due to higher MSE values and lower *R*^2^ scores, hence demonstrating unacceptability within such complex geospatial and temporal earthquake data. SeismoQuakeGNN has now proved itself to be a better model, having a real handle on both spatial and temporal information, thus being a new leap forward in forecasting earthquakes. Future studies may, for example, consider multimodal data integration of waveform signals and textual records to improve the predictive capacity of high-magnitude seismic events.

## Data Availability

The original contributions presented in the study are included in the article/supplementary material, further inquiries can be directed to the corresponding author.
